# Molecular pathways enhance drug response prediction using transfer learning from cell lines to tumors and patient-derived xenografts

**DOI:** 10.1038/s41598-022-20646-1

**Published:** 2022-09-27

**Authors:** Yi-Ching Tang, Reid T. Powell, Assaf Gottlieb

**Affiliations:** 1 Center for Precision Health, School of Biomedical Informatics, University of Texas Health Science Center at Houston, Houston, TX 77030 USA; 2 Center for Translational Cancer Research, Texas A&M University, Houston, TX 77030 USA

**Keywords:** Cancer genomics, Computer modelling, Medical research, Translational research

## Abstract

Computational models have been successful in predicting drug sensitivity in cancer cell line data, creating an opportunity to guide precision medicine. However, translating these models to tumors remains challenging. We propose a new transfer learning workflow that transfers drug sensitivity predicting models from large-scale cancer cell lines to both tumors and patient derived xenografts based on molecular pathways derived from genomic features. We further compute feature importance to identify pathways most important to drug response prediction. We obtained good performance on tumors (AUROC = 0.77) and patient derived xenografts from triple negative breast cancers (RMSE = 0.11). Using feature importance, we highlight the association between ER-Golgi trafficking pathway in everolimus sensitivity within breast cancer patients and the role of class II histone deacetylases and interlukine-12 in response to drugs for triple-negative breast cancer. Pathway information support transfer of drug response prediction models from cell lines to tumors and can provide biological interpretation underlying the predictions, serving as a steppingstone towards usage in clinical setting.

## Introduction

Cancer cell lines have been designed to serve as in-vitro research models to facilitate high-throughput screening of cancer drugs. However, cancer cell lines have been shown to be poor predictors of human responses owing to factors such as underrepresented tumor heterogeneity and lacking components of the tumor microenvironment^1^. Facing these shortcomings, there has been a shift towards tailoring drug response profiling using in-vivo tumors or patient-derived xenografts (PDX)^1^. However, the process of deriving PDX remain costly and time consuming, which hinders its application into clinical practice. Thus, a computational framework that would leverage the plethora of knowledge and data already collected on cancer cell lines to bridge the gap between cell lines and in-vivo tumors or PDX data may be useful in addressing the cost and time limitations of PDX.

Using data from cancer cell lines has the potential to enrich the currently scarce tumor data. However, there are three additional challenges facing computational methods that aim to translate models developed on cell lines to tumor data: mapping genomics data from in-vitro to in-vivo measured data, mapping outcome measures and explaining the predictions.

The first challenge involves the differences in collected pre-treatment genomic data. Pre-treatment or baseline gene expression data are effective biomarkers for predicting drug response of cell lines, compared to gene-level mutation data, copy number variation data^2^. However, estimation of gene expression level may vary between platforms or between batches, leading to poor reproducibility^3^. In order to address this limitation, Geeleher et al^4^ introduced a robust statistical correction methodology, showing that cell line model can guide the prediction of clinical drug sensitivity when they have similar distributions, with Gruner et al^5^ successfully applying their regression model to cancer sub-types of Triple- Negative Breast Cancer (TNBC) patient tumors. Additionally, Turki, Wei, and Wang^6^ augmented the cell line training data by including the best matching tumor gene expression subsets through the Procrustes analysis.

A second challenge is the mapping of aggregates extracted from drug response curves in cell lines, such as IC50 or Area under the drug response curve (AUC) to dichotomous values required in making treatment decisions in patients, such as responsive/non-responsive. In order to address this challenge, a decision model developed by Cheng et al^7^ first selected the most similar cell lines for each patient and then prioritized therapeutic agents based on the AUC on the most similar cell lines. Another approach by Sharifi-Noghabi et al^8^ used an ad-hoc threshold to binarize IC50 of cell lines and trained a classifier incorporating embedded multi-omics data to classify clinical outcomes.

The final challenge involves model explainability, which is important to achieve better clinical utility^9^.

In this study, we describe a prediction model that addressed these three challenges. We leverage large-scale cancer cell line data and biological knowledge in the form of molecular pathways to transfer models trained on cell lines in order to predict outcomes in in-vivo tumor and ex-vivo patient-derived xenograft (PDX) data. We address the transferability of pre-treatment transcriptomics by mapping them to molecular pathways, which has proved robust in our previous publication Tang and Gottlieb^10^ and by identifying pathways with large effect on the prediction, we increase the explainability of the models, providing mechanistic interpretation of the sensitivity or resistance of a drug on a certain tumor.

The use of large-scale cancer cell line data through transfer learning enable us to address the limited availability of tumor data and has proven beneficial for transferring different modalities^6^, tissues^11^ or data sources^12^. In addition, we integrated both genomic and chemical properties of drugs along with pre-treatment gene expression to enable pan-cancer, pan-drug predictions, which has been shown to be beneficial in improving generalizability comparing to previous single-drug models^13^.

Our transfer learning framework enables us to provide predictions both to dichotomous outcomes (responsive or not) and to continuous outcomes (AUC on PDX data), thus addressing the second challenge, involving translating response curves to more clinically-meaningful outcomes. In order to demonstrate this, we validated our transfer learning model in three independent scenarios: in in-vivo tumors data from clinical trials and in ex-vivo PDX data with both dichotomous and continuous outcomes. To conclude, we demonstrated that knowledge of in-vitro cell line drug response can be beneficial for inferring drug sensitivity in tumors and that biologically informed computational model can predict drug sensitivity and provide system biology insights about drug actions.

## Results

### Prediction accuracy with transfer learning

We considered clinically relevant data across two axes. The first axis is the data type, including human in-vivo tumor drug screen data and ex-vivo PDX data. The second axis is whether the response was measured as dichotomous (responsive/non-responsive) or continuous (Area under the drug response curve, AUC), which resulted in three scenarios across the two axes: tumor data, consisting of 24 drugs and 16 cancer types, PDX with dichotomous outcomes (PDX-D) consisting of 22 drugs and six cancer types measured in PDX models, and PDX with continuous outcomes (PDX-C) which is a screening of 340 drugs in triple-negative breast cancer (TNBC) measured in PDX models (Methods, Tables 1, S1–S2).

**Table 1:** Datasets used in this study.

	GDSC Cell lines	Transfer learning to data source type (outcome type)		
		Tumors (Dichotomous)	PDX (Dichotomous)	PDX (Continuous)
Sample size	98,557	517	256	4,641
Number of drugs	142	24	22	340
Number of cancer types	31	16	6	1

**Figure 1:**
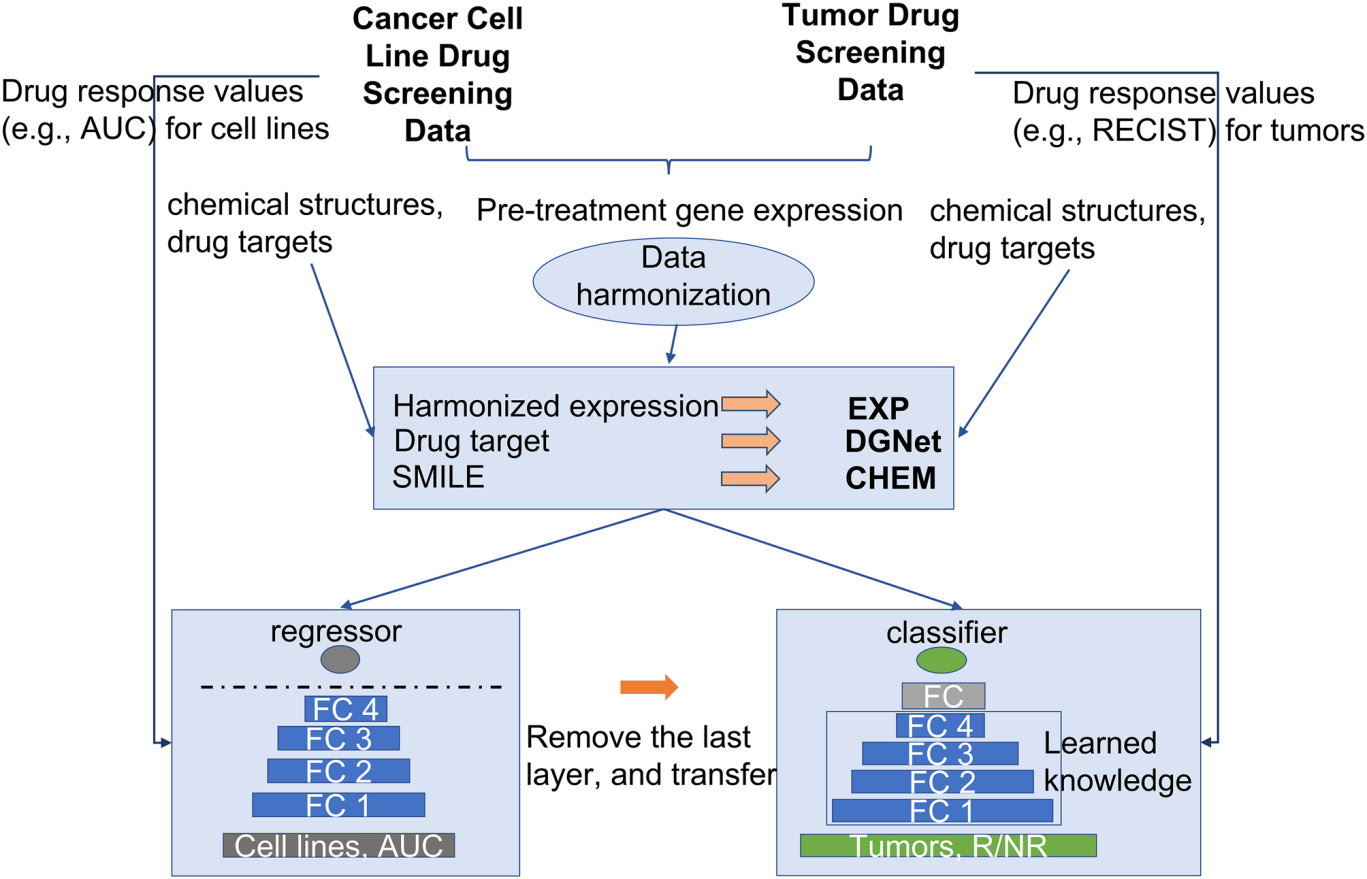
Workflow of the transfer learning model. From top to bottom, pre-treatment cell line and tumor gene expression levels were harmonized. Genomic and chemical features were generated respectively. Cell line data were then used to pretrained a cell line model, while tumor data were feed into a new model which was trained with the pretrained model.

Our pre-trained model is a deep neural network trained on the GDSC cancer cell line data (Methods). In our previous study, we demonstrated high prediction performance of our model on cancer cell line data, outperforming previous methods^10^. We applied our transfer learning method to the three scenarios following the workflow depicted in Fig. 1. For each dataset, we compared the transfer learning model to its corresponding model which has no transferred layers. We also compared different sets of pathway features, including the Pathway Interaction Database (PID) pathways^14^, REACTOME pathways^15^, and the combination of both, to test the contribution of these pathways to performance. In the following sections, we analyze each of the three scenarios separately.

**Figure 2:**
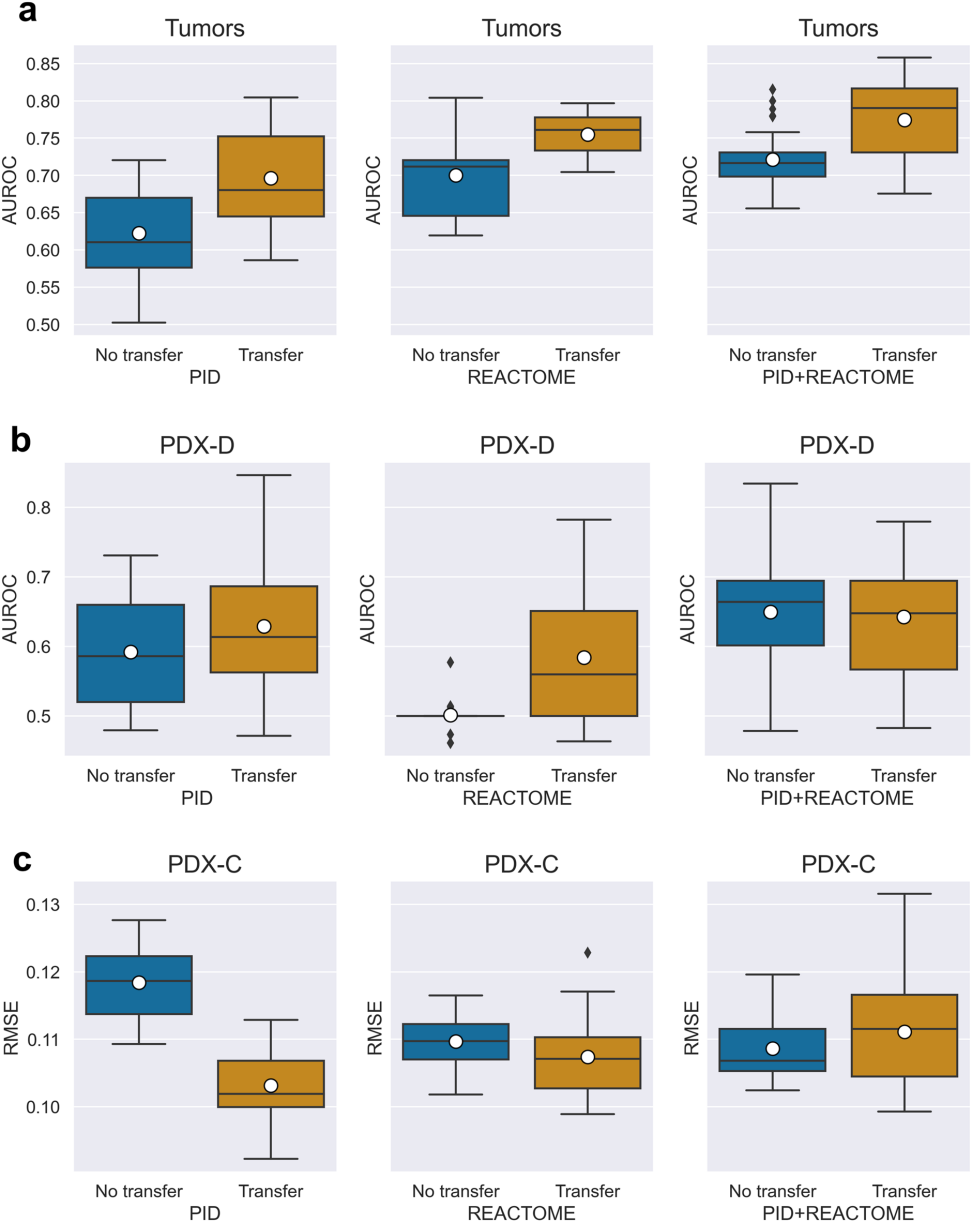
Box plot of performance score distribution across repetitions of cross validation. Brown boxes are transfer learning models, and blue boxes are models without transferred layers (n = 25 scores per model for each box). White circles are mean lines. (**a**) and (**b**) are the Area Under the Receiver Operating Curve (AUROC) for the tumor dataset, and the PDX-D datasets, respectively. (**c**) the Root Mean Squared Error (RMSE) obtained from the PDX-C dataset.

The result of five repeats of five-fold cross validation showed that the best AUROC is 0.77 for the tumor data and 0.64 for PDX-D. For the PDX-C prediction, the average RMSE was 0.11 (Tables S3–S4, Figs. 2, S1). The best performance in the dichotomous predictions (tumor and PDX-D) was obtained when combining both PID and REACTOME. In the Tumor data, we obtained better performance when using transfer learning than without transfer learning (Mann–Whitney U test *p* value < 0.002), but for PDX-D, the un-transferred model obtained slightly better results. For the predictions of PDX-C, there was minor difference in performance between using only PID, only REACTOME or both, with slightly better performance when using the PID pathway. Here too, transferred models were better than non-transferred models (Mann–Whitney U test *p* value < 2E-09). As such, we used the best performing model of each dataset for further analysis (Tables S3–S4).

### From cell lines to tumor data

**Figure 3:**
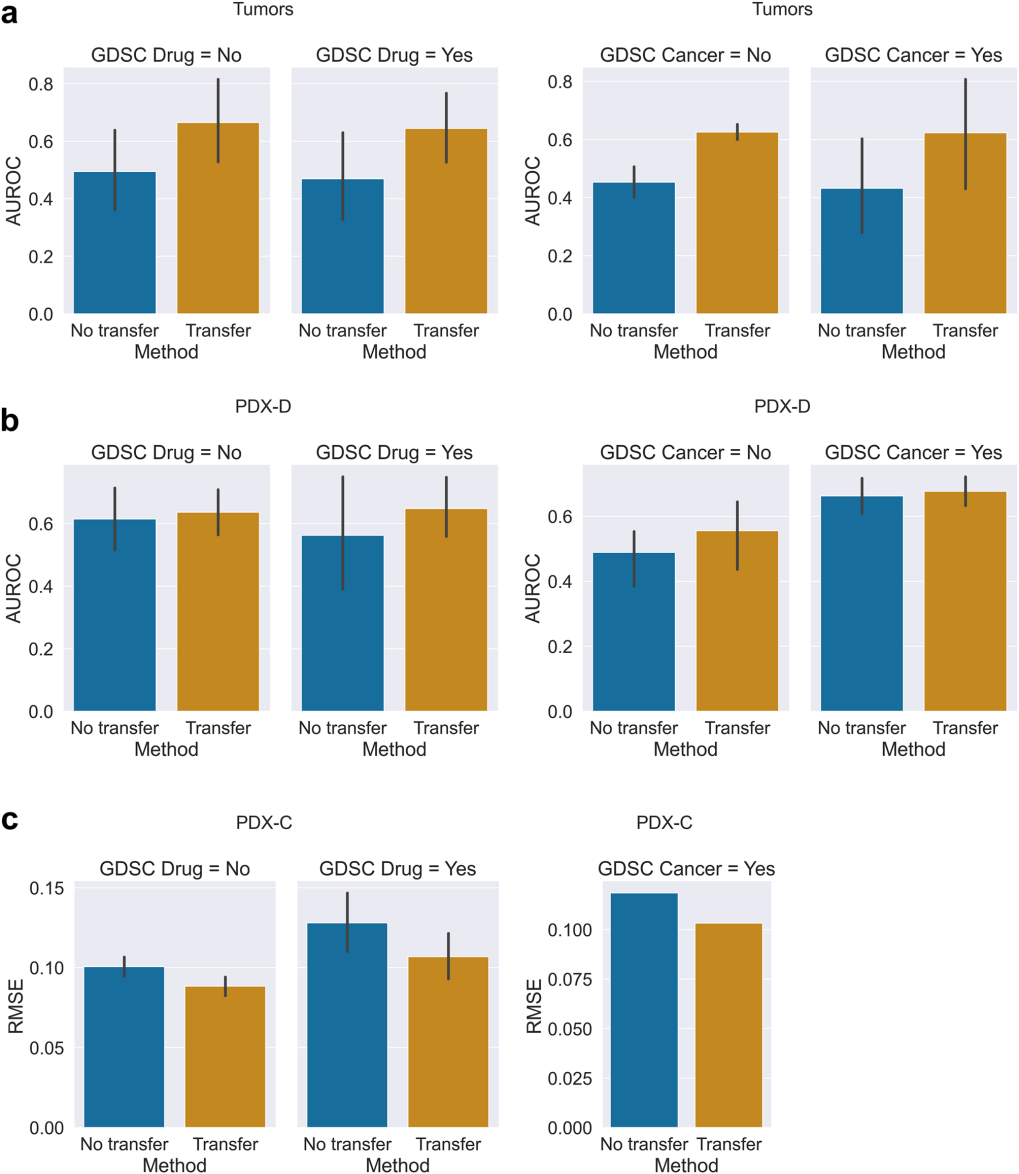
Bar plot of per-drug and per-cancer performance averaged across drugs (left panel) and cancers (right panel). Comparison is between predictions made on drugs or cancer types that exist in the cell line repository of GDSC (panels titles “GDSC Drug = Yes” or “GDC cancer = Yes”) and drugs/cell lines that appear only in the tumor or PDX samples (panels titles “GDSC Drug = No” or “GDC cancer = No”). Bar lengths are mean values, and error bars represent one standard deviation. (**a**) Area Under the Receiver Operating Curve (AUROC) for the tumor data averaged across 15 drugs (n = 8, 7 for GDSC drugs and non-GDSC drugs), and 10 cancers (n = 8, 2 for GDSC cancer types and non-GDSC cancer types). (**b**) The average of AUROC over the PDX-D predictions consisting of 12 drugs and 5 cancer types (n = 3, 9 for GDSC drugs and non-GDSC drugs; n = 2, 3 for GDSC cancer types and non-GDSC cancer types). (**c**) The average Root Mean Squared Error (RMSE) for 340 drugs and 1 cancer type in the PDX-C predictions (n = 30, 310 for GDSC drugs and non-GDSC drugs, n = 1 for GDSC cancer types).

The tumor data was assembled from multiple sources (Methods). The best performance in a five-fold cross validation was achieved by incorporating both PID and REACTOME pathways (AUROC of 0.77 ± 0.06, Fig. 2a and Table S3). Additionally, we compared the contribution of transferred learning to per-drug (across all tumors treated by the drug) and per-cancer (across all drugs treating that cancer type) prediction. Transfer learning improved the AUROC by 49.8% and 47.8% relative to not using transfer learning (on average) for the per-drug and the per-cancer predictions, respectively (Fig. 3a).

Some of the drugs tested in the in-vivo studies were not tested in the in-vitro panel of GDSC. The result of within-sample comparison showed that there is a small difference between drugs/cancer types included in the in-vitro cell line panel (0.65 ± 0.2 mean AUROC for drugs in GDSC vs. 0.66 ± 0.2 mean AUROC in the set of drugs not in GDSC; 0.62 ± 0.3 mean AUROC for cancer types in GDSC vs. 0.63 ± 0.03 mean AUROC across cancer types not in GDSC, Fig. 3a and Table S5).

We highlight one example from our prediction results: our model obtained AUROC of 0.9 ± 0.001 for predicting sensitivity to everolimus, an immunosuppressive drug of type mTOR inhibitor, screened for breast cancer patients in our data. Our top eight predictive pathways (using Shapley values) display significantly different level of enrichment between responders and non-responders to everolimus (Benjamini–Hochberg FDR adjusted *p* < 0.05, Table S6). These pathways are associated with mTOR pathways in different ways. For example, one of the top pathways, Reactome’s VESICLE MEDIATED TRANSPORT (FDR-adjusted *p* value < 6E^−4^, Table S6), involves ER-to-Golgi transport machinery that co-regulates with the mTOR proteins in proliferation and growth of breast cancer cells^16–18^. Furthermore, another predictive pathway, Reactome’s CHEMOKINE RECEPTORS BIND CHEMOKINES (FDR-adjusted *p* value < 6E^−4^), has a reported role in the mTOR function in both immune cells and tumor cells through the pairing of chemokine to its receptor^19,20^. Notably, chemokine CXCL12, one of the components in the predicted pathway, is a mTOR-associated chemokine in breast cancer^20^. It has been further demonstrated that there is a positive feedback loop between mTOR activation and the binding of CXCL12 to its receptor CXCR4—inhibiting the mTOR activity may prevent cancer metastasis because it subsequently decreases CXCL12 interaction.

**Figure 4:**
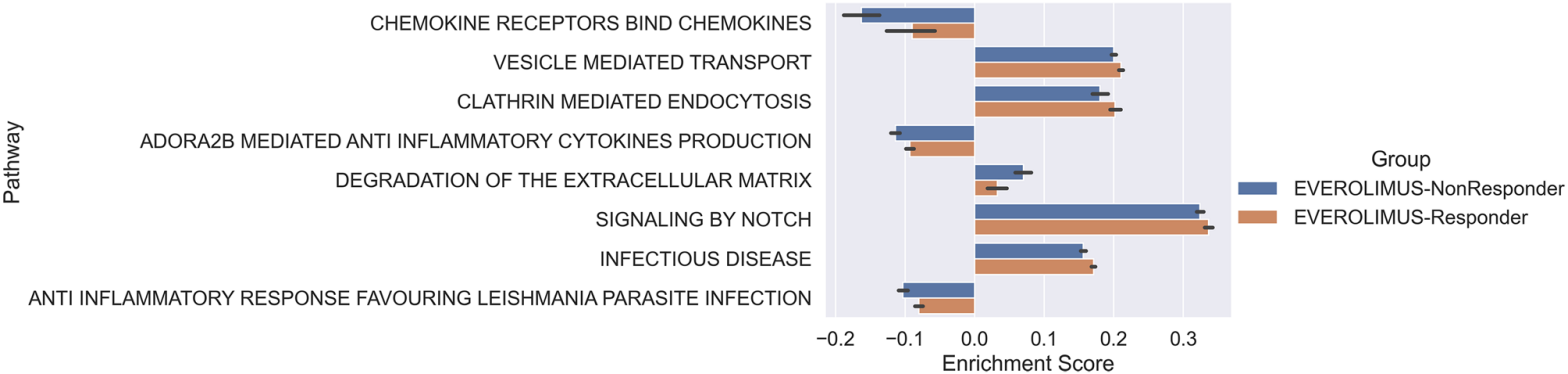
Top pathways contributing to prediction of everolimus treatment outcomes that are also significantly different between everolimus responders and non-responders.

In addition, our finding showed that a specific anti-inflammatory response (Reactome’s ANTI INFLAMMATORY RESPONSE FAVORING LEISHMANIA PARASITE INFECTION, FDR-adjusted *p* value < E^−4^) is the key linking to resistant response. This pathway has a positive control on the expression of host mTOR^21^. Highly active the pathway signals, higher expression levels of mTOR proteins. Our result showed that non-responder samples had downregulated expression in the pathway, indicating there is a low expression level of mTOR signals in non-responder samples (Fig. 4). Moreover, signals related to this pathway have been associated with drug resistance (or poor clinical outcomes) in breast cancer. For example, CD163-mediated anti-inflammatory response^22,23^, Adenosine receptor 2B (ADORA2B) mediated anti-inflammatory cytokines production^24^ and IL10 synthesis that plays a role in immune escape^25^. Together, our finding supports the current knowledge that cellular activity in the mTOR signaling pathway might affect the drug sensitivity in tumors.

### From cell lines to PDX with dichotomous outcomes

The ex-vivo PDX-D drug screen dataset was obtained from Gao^21^, which used modified RECIST criteria for a dichotomous drug response. The PDX-D dataset is highly imbalanced with 64 responders and 1628 non-responders, and the number of non-responders per drug is 4–43 times larger than the number of responders. To address this imbalance, we subsampled from the non-responders (see Methods). In this case, the best AUROC (0.63 ± 0.11) was obtained when integrating the PID pathways only (Table S3).

Our transfer learning approach was beneficial for both drugs that are included in GDSC and drugs not included in it, where AUROC of the drug included in GDSC increased from 0.60 ± 0.2 (without transfer learning) to 0.68 ± 0.2 (with transfer learning), while for drugs not in GDSC improved from 0.60 ± 0.2 to 0.63 ± 0.1. Similarly, the improvement in the cancer types appearing in GDSC improved from 0.66 ± 0.05 to 0.68 ± 0.04 and those not appearing in GDSC improved from 0.49 ± 0.07 to 0.56 ± 0.01 AUROC (Fig. 3b and Table S4).

We compared per-drug AUROC for three drugs that appear in our dataset and were also reported in previous studies^4,13^, including paclitaxel, gemcitabine and erlotinib. Our pathway-based transfer learning model achieved better performance on paclitaxel and erlotinib with 0.8 ± 0.1 and 0.67 ± 0.16 AUROC, respectively but achieved lower performance on gemcitabine than Geeleher et al^4^ (AUROC of 0.53 ± 0.09 vs. 0.59, Table 2). We note that one of the compared methods, MOLI^13^, showed improved performance by incorporating mutation and copy number variation data on both gemcitabine and erlotinib. For the purpose of comparing with the multi-omics models of MOLI, we incorporated mutations and copy number variations to our pathway-based model. As a result, our multi-omics model achieved an AUROC of 0.83 ± 0.2 for erlotinib, improving upon the best MOLI model but did not improve on gemcitabine (MOLI complete pan-drug multi-omics, Table S7).

**Table 2:** Methods comparison based on area under the receiver curve (AUROC) of three drugs in the PDX-D dataset. The best performance is in bold.

Drug	Our model	Geeleher et al., Expression	MOLI complete, Expression^a^
Paclitaxel	**0.80 ± 0.1**	0.52	0.69
Gemcitabine	0.53 ± 0.09	**0.59**	0.52
Erlotinib	**0.67 ± 0.16**	**0.67**	0.39

^a^The performance scores were adopted from Sharifi-Noghabi (2015)’s paper.

### From cell lines to PDX with continuous outcomes

The ex-vivo PDX-C drug screen dataset was obtained from Powell^22^, that reported normalized dose-dependent AUC. We framed the PDX-C as a regression problem. We tested two scenarios: one using the original GDSC drug response curve AUC and one by adjusting the GDSC AUC calculation to match the PDX data AUC calculation. (Methods). Our transfer learning approach achieved good performance using both drug AUC measures, but the model using the adjusted AUC values displayed higher benefit from transfer learning (Fig. S1). Our model incorporating PID pathways obtained RMSE of 0.1 ± 0.005 in cross validation, which is only slightly lower than when using REACTOME alone or the combination of both (Table S4). There is a small improvement (RMSE of 0.02) over model trained without transfer learning (Fig. 3c and Table S4).

We exemplify a PDX from a specific PDX with high correspondence between the observed drug sensitivity and the model predicted one. Our model obtained a high correlation between predicted drug response and actual response values with R^2^ = 0.86, Pearson’s ρ = 0.93 for PIM137 (Fig. S2). Our model identified two pathways that are correlated with individual variation in drug response, which are PID’s HDAC_CLASSII_PATHWAY (Pearson’s ρ = 0.12, *p* value < 4E^-16^) and PID’s IL_12_2PATHWAY (ρ = 0.1, *p* value < 4E^-10^). Both pathways play a role in the immune response. Histone deacetylases (HDACs) regulates enzyme activity responsible for inflammatory development while interleukin 12 (IL-12) is responsible for balancing autoimmune attack and immune response against foreign antigens^26,27^. Class II HDAC inhibitors have shown efficiency in reducing cell proliferation in breast tumors, both in-vivo and in-vitro^28^. Indeed, a previous study showed improved immune responses in TNBC patients treated with combination of HDAC inhibitor and immune checkpoint inhibitor^29^. IL-12 also has well-documented in-vivo and in-vitro antitumor activity^30^. Through promoting antitumor immunity that activates natural killer (NK) and T cells, IL-12 demonstrates potential potency in combination with immunotherapy. For example, TNBC patients that were unresponsive became sensitive to anti-PD-L1 immunotherapy after injecting IL-12^31^. The combination of HDAC inhibitor and immunocytokine NHS-rmlL12 improved protective immunity in breast tumors that have inherited immune deficiency^32^.

We note that the remaining eight out of top ten pathways for PIM137 across all tested drugs were features derived from the molecular fingerprints of the drug structure.

## Discussions

We have developed a new transfer learning model for predicting drug sensitivity in pre-treatment in-vivo and ex-vivo tumor data based on mapping of pre-treatment genomic data to molecular pathways. Our approach utilized a model pre-trained on large-scale cancer cell line drug screening data and incorporates both genomic and chemical features. We demonstrated that our pathway-based method achieved good performance in different scenarios, including in-vivo tumor data and ex-vivo PDX data in a cross-validation and had better, or comparable performance to previous expression-based models for specific drugs. Our approach proved especially robust when dealing with tumor data from different sources and using different measures to determine drug sensitivity.

The proposed approach supports a flexible model architecture that is adaptable to data representation from one task to other related tasks, enabling information extraction directly from cell line response without continuous-discrete conversion. We showed that in general, knowledge transferred from cell lines improves prediction accuracy in tumor or PDX drug sensitivity data. The only exception was in the dichotomous PDX data, where transfer learning did not manage to improve the results. A possible reason is that the PDX-D data shared the least number of drugs and cell types with the GDSC cell line data. Only 3/12 drugs and 2/5 cell lines in the PDX-D data exist also in GDSC (Table S5). In contrast, 8/15 of the drugs and 8/10 of the cell lines in the tumor data appear also in GDSC, where the difference between transferred and untransferred models was to most profound. In PDX-C, only 30/340 drugs were in GDSC, but the only cell type used appears also in GDSC, suggesting that existence of shared cancer cell types has larger influence on the improvement of the transfer learning than the number of shared drugs.

One strength of our approach is the explainability of our model via the functional roles of molecular pathways. Pathway-based features can reflect molecular interactions between genes active in tumors and the downstream effect of drug-targets, as we exemplify for everolimus.

There are three major limitations of our method. First, the use of Shapley values and statistical significance infers associations, not causal relation. Experimental validation is thus needed in order to further identify the possible roles the top predictive pathways play in drug sensitivity/resistance. Second, we have used only transcriptomic data in our study, owing to lack of other genomic data from the studies we assembled. This enabled our models to transfer learn predictions for hundreds of drugs across multiple types of cancer, which has previously limited methods relying on multi-omics data. Thus, the higher prevalence of transcriptomic data in clinical cancer studies together with the observation that transcriptomic data has been proven to be the best predictor of drug response^2^ supports a practical direction towards clinical application. However, there might be cases where other types of genomics data help obtain a significant performance of the models. Two such examples are responses to gemcitabine and erlotinib. Since our method is capable of incorporating other types of genomic data, such as mutation and copy number variation data, we demonstrate that we are able to improve significantly our performance on erlotinib through the incorporation of these types of genomics data. However, we failed to improve upon previous methods for gemcitabine, either with only transcriptomic data or with multi-omics approach. One possible explanation is the existence of specific mutations in TP53 that were shown to be predictive of gemcitabine sensitivity in a clinical trial^33^ and might have not have been as well represented in our pathways as in methods relying on individual genes. Finally, in this study, we focused on monotherapy prediction and excluded drug combination data. While we previously presented a pathway-based deep neural networks to predict in-vitro drug combination^34^, the large space of drug combination and the relative scarcity of in-vivo drug combination screens limit the transferability of our models in this scenario.

A noticeable trait of our method is that performance was largely dependent on sufficient sample size, such that our method tended to perform much better on drugs and cancer types that had relatively large sample size. This is evident from the fact that the overall AUROC is much higher than the average on the drugs/cancer types as the overall AUROC is influenced more from drugs/cancer types with large sample size while averaging on the individual performance of each drug/cancer type assigns equal weight to drugs/cancer-types regardless of sample size. Similarly, we obtained relatively lower performance on the PDX-D dataset, owing in part to the low number of the responders within the dataset. It is thus imperative to continue to conduct more experiments on underrepresented drugs/cancer-types in order to improve the prediction power of computational methods like the one we presented.

## Conclusion

We demonstrate that through a pathway-based transfer learning model, we are able to transfer information from large compendia of cancer cell line data to in-vivo and ex-vivo data. This is yet another steppingstone in the move from research to clinical application, where our model may be potentially used as a pre-screening tool for patients.

## Materials and methods

### Data

In-vitro human cell lines screen was obtained from the Genomics of Drug Sensitivity in Cancer (GDSC)^35^. We filtered cell lines that had no gene expression profile or no measured drug response value. Human tumor drug screening datasets were collected from literatures^36–39^ and GEO search using the same keyword searching strategy that was described Lee et al. paper^39^ with the following search terms: “pre-treatment OR baseline transcriptome AND cancer patient survival”, “pre-treatment gene expression AND cancer drug response” performed in November 2021. We considered only tumor samples that had available pre-treatment gene expression and drug response and patients that have been treated with only one drug during the treatment period in order to avoid combinatorial effect. Most in-vivo studies use the Response Evaluation Criteria in Solid Tumors (RECIST) criteria to categorize clinical drug sensitivity (i.e., responder, non-responder), while some measures were based on the time from treatment to disease progression, such as Progression Free Survival (PFS), Recurrent Free Survival (RFS) and Time to Progression (TTP) (Table S1). Ex-vivo PDX data was downloaded from Gao et al^36^ (dichotomous response) and for primary treatment-naïve triple-negative breast cancer from Powell et al^38^ (AUC measured response). Notably, the Powell *et* al. dataset was aimed at drug repurposing and thus tested a panel of FDA approved agents, including drugs not intended for cancer, with the hope of finding new drug mechanism of action and shorten the translational time line. We downloaded PID^14^ and REACTOME^15^ pathway gene sets from MSigDB^40,41^.

### Ethical approval

All data used in this study were downloaded from publicly available repositories and require no additional institutional ethical approval.

### Preparing pathway-based gene expression features

Harmonization: To make expression values generated from different platforms comparable (Table S1), all data were log-transformed and harmonized. We selected common gene set in cell line gene expression data and tumor gene expression data to perform data harmonization following previously published procedures for batch correction^4,42^. In short, each dataset was treated as a batch, and then was standardized gene-wise to have a mean of zero and standard deviation of one. Random effects across batches were then estimated via the Bayesian method developed by Johnson et al^42^. Third, scaled data was adjusted based on the estimators obtained from the previous step. All three steps were completed by using the pyCombat package.

Pathway enrichment analysis: We conducted single-sample Gene Set Enrichment (ssGSEA) analysis on the harmonized gene expression data to quantify pathway activity in each sample using the PID and REACTOME pathways^14,15^. We ran permutation tests 1000 times using the python library GSEApy to establish significance scores. We termed the resulting pathway features as EXP.

### Preparing drug features with chemical structure and target information

Drug features were represented by canonical SMILES and drug targets, respectively. We used one-hot vectors with a fixed size of 256 bits of Morgan fingerprint to encode chemical structure for each drug (CHEM features). For drug target data, we performed the Network-based Pathway Enrichment Analysis (NetPEA)^43^ with PID and REACTOME pathways^14,15^ to quantify the propagated effects of therapeutic targets on pathway genes within the protein–protein interaction network which was downloaded from the STRING database^44^. Detailed procedures for both feature types were described in our previous work^10^ (DGNet features).

### Building pre-trained cell line model using in-vitro drug sensitivity data

After data preprocessing described above, input features consisting of CHEM, DGNet and EXP were concatenated and fed into a deep neural network with four hidden layers with 1000, 800, 500, and 100 neurons respectively. Dropout was applied to the neuron level with 10% probability to drop as described in previous studies^10^. Other hyperparameters were also adopted, including learning rate of 0.0004, early stopping at 30 patience (the number of epochs to wait before early stop if no progress on the validation set), ELU activation function for each hidden layer except for the output layer, and the weight values after a gradient update were clipped between −5 and 5. The loss function is RMSE, and the optimization function is Adamax. We performed five-fold cross validation and then used the best model to refit the whole dataset to obtain a pre-trained cell line model. The drug sensitivity data in the GDSC dataset were quantified using two continuous variables, IC50 and AUC, representing half maximal inhibitory concentration and the area under the fitted dose response curve, respectively. We converted IC50 to -log_10_IC50 and use 1-AUC to define drug sensitive as the AUC values ranges from 0 to 1^45^. We eventually used only the model that predicts 1-AUC as the final pre-trained model because it had better performance in terms of Root Mean Squared Error (RMSE) on the GDSC data, with an average RMSE of 0.096 across five test folds, comparing to mean RMSE of 0.42 achieved by the model that used − log_10_IC50 as the response variable.

### Fine-tuning the transfer learning model

We considered different parameter settings for the pretrained model, including the number of pre-trained layers to transfer and whether to retrain the pretrained parameters or no retraining at all. We also investigated several hyperparameter spaces, including the choices of optimizer, activation function, batch size, dropout rate, learning rate, the number of neurons for the newly added layers, and the number of new hidden layers. The range of parameter values explored is detailed in Table S8. The best set of parameters was determined using the Bayesian optimization via the BayesianOptimization python library. The final parameters used for model comparison are listed in Table S9.

### Transfer learning from cell lines to tumors

Harmonized expression levels from the GDSC cell lines and the *tumor/PDX* datasets were converted into pathway-level features following the procedure described above. Drug features were obtained using the same processing procedures. Features from the pre-trained cell line in-vitro model were used to fit a deep classifier (Fig. 1). Hyperparameters were fine-tuned as outlined above. The sigmoid function was used in the output layer to generate prediction in the form of probability. Before training, clinical responders were coded as 1 while non-responders were coded as 0. During training, binary cross entropy was optimized based on training and validation data. The model performance was evaluated by the rest of test set. We performed five-fold cross validation for five times and averaged 25 folds the area under the receiver operating curve (AUROC) for model comparison.

### Transfer learning from cell lines to PDX

We tested our transfer learning models on two types of PDX outcomes: dichotomous (responsive/non-responsive) and continuous AUC.

For the dichotomous outcome (PDX-D), we designed a classification task that used the same workflow described for the tumor transfer learning model, but due to the large class imbalance between responders and non-responders, we randomly subsampled from the non-responder class, so that every drug has a ratio of 1:3 for responders and non-responders. This ratio achieved the best performance relative to compared ratios of 1:2, 1:3 and 1:4, achieving mean AUROC 0.58, 0.61, 0.58, respectively.

For the PDX-C regression task, we used mean squared error (MSE) as the loss function, and no activation function was used in the output layer to restrict output range. Root mean squared error (RMSE), averaged over five folds, was used to evaluate model performance. Other procedures such as expression data harmonization, feature engineering, pretrained model construction, transfer learning model tuning, and validation were also performed following the same practice as detailed for tumors. This dataset used a customized AUC measures which has an inverse relationship with the dose-dependent AUC of GDSC (Fig. S3). Therefore, the higher the adjusted AUC value, the more sensitive to a drug. In order to generate comparable AUC values between the two studies, we downloaded the raw drug screening data from GDSC (ftp://ftp.sanger.ac.uk/pub/project/cancerrxgene/releases/current_release/GDSC2_public_raw_data_25Feb20.csv) and applied the same statistical workflow as previously described^38^.

### Compare between-sample and within-sample performance

We compared prediction performance with/without using the transfer learning method by averaging AUROC scores for dichotomous predictions, and RMSE values for continuous outcomes. For within-sample comparison, we further broke down samples into GDSC drugs/GDSC cancer types and non-GDSC drugs/non-GDSC cancer types. We focused on drugs/cancer types that have at least five samples to calculate performance scores on a single drug/single cancer type basis. Finally, we averaged scores across GDSC samples, and non-GDSC samples, respectively for comparison.

### Explaining prediction outcomes by molecular pathways

We estimated the contribution of input features to the prediction values based on Shapley values^46^ via the DeepExplainer function of the SHAP^47^ package. Feature importance is quantified by the mean of absolute Shapley values across samples. The higher the value, the greater contribution to the prediction.

For dichotomous predictions, we used true positives and negatives to define responders and non-responders, respectively as they are accurately labeled by our models. After prediction categorization, we identified top contributing pathways for two groups of samples, respectively.

For continuous predictions, we ordered features by ranked importance across all samples. Enrichment of each pathway was compared for two groups using Mann–Whitney U test, and the corresponding two-sided p values were adjusted with Benjamin-Hochberg false discovery rate method. We set the significance level at 0.05 for the FDR-adjusted *p* values.

### Statistical analysis

Results of repeated cross validation are presented in the format of mean ± SD. Statistical tests, significance, and the number of samples are presented followed by the respective data or figure legends.

## Supplementary Information

Supplementary Information 1.

Supplementary Information 2.

Supplementary Information 3.

## Data Availability

Sources of raw drug screening data are described in this manuscript. Processed data (molecular fingerprint, pathway enrichment scores for drug targets and pre-treatment gene expression) are available at https://doi.org/10.5281/zenodo.6093818.

## Code availability

Python code that are critical to the analysis and generation of figures are available at https://github.com/TangYiChing/TranPathDSP.
