# Supplementary material for: Molecular pathways enhance drug response prediction using transfer learning from cell lines to tumors and patient-derived xenografts
Source: Sci Rep. 2022 Sep 27;12:16109. doi: 10.1038/s41598-022-20646-1 (PMC9515168; doi:10.1038/s41598-022-20646-1)
Supplement: Supplementary file 1 — Supplementary Information 1. [file 41598_2022_20646_MOESM1_ESM.pdf]

Per-drug performance for tumor data

Name	GDSC drug	N	Transfer AUROC	Transfer Std.	Transfer CI 95%	No transfer AUROC	No transfer Std.	No transfer CI 95%
ANASTROZOLE	No	51	0.61	0.0009	[0.61, 0.61]	0.65	0.05	[0.59, 0.71]
EVEROLIMUS	No	88	0.90	0.0009	[0.90, 0.90]	0.82	0.03	[0.78, 0.87]
EXEMESTANE	No	16	0.58	0	-	0.45	0.1	[0.28, 0.62]
FLUOROURACIL	No	11	0.33	0	-	0.18	0.2	[-0.01, 0.36]
LETROZOLE	No	99	0.61	0.001	[0.61, 0.61]	0.53	0.03	[0.49, 0.57]
TRASTUZUMAB DERUXTECAN	No	48	0.62	0	-	0.50	0.05	[0.44, 0.56]
VEMURAFENIB	No	8	1.00	0	-	0.33	0	-
AFATINIB	Yes	19	0.57	0.006	[0.57, 0.58]	0.38	0.08	[0.28, 0.48]
CISPLATIN	Yes	20	0.53	0.01	[0.51, 0.54]	0.31	0.06	[0.24, 0.39]
DABRAFENIB	Yes	25	0.41	0.004	[0.41, 0.42]	0.34	0.03	[0.30, 0.38]
GEMCITABINE	Yes	12	0.51	0	-	0.32	0.04	[0.27, 0.37]
PACLITAXEL	Yes	5	0.67	0	-	0.40	0.09	[0.29, 0.51]
SORAFENIB	Yes	68	0.89	0.01	[0.87, 0.90]	0.89	0.04	[0.84, 0.93]
TAMOXIFEN	Yes	24	0.76	0.02	[0.74, 0.79]	0.74	0.04	[0.69, 0.78]
TEMOZOLOMIDE	Yes	7	0.83	0	-	0.32	0.1	[0.18, 0.45]

Per-drug performance for PDX-D dataset

Name	GDSC drug	N	Transfer AUROC	Transfer Std.	Transfer CI 95%	No transfer AUROC	No transfer Std.	No transfer CI 95%
BGJ398	No	12	0.53	0.10	[0.41, 0.64]	0.40	0.14	[0.23, 0.57]
BINIMETINIB	No	64	0.71	0.05	[0.64, 0.78]	0.73	0.05	[0.66, 0.79]
BKM120	No	32	0.48	0.07	[0.39, 0.56]	0.48	0.06	[0.40, 0.55]
BYL719	No	16	0.73	0.16	[0.53, 0.93]	0.73	0.14	[0.55, 0.90]
CGM097	No	12	0.81	0.14	[0.63, 0.99]	0.83	0.22	[0.56, 1.10]
DACARBAZINE	No	8	0.57	0.09	[0.45, 0.68]	0.58	0.10	[0.46, 0.71]
ERLOTINIB	No	12	0.67	0.16	[0.48, 0.87]	0.70	0.09	[0.59, 0.82]
GEMCITABINE	No	28	0.53	0.09	[0.42, 0.65]	0.45	0.10	[0.33, 0.57]
HDM201	No	8	0.62	0.16	[0.41, 0.82]	0.47	0.23	[0.19, 0.75]
LEE011	Yes	24	0.66	0.06	[0.58, 0.73]	0.71	0.08	[0.61, 0.82]
PACLITAXEL	Yes	20	0.80	0.10	[0.68, 0.92]	0.77	0.15	[0.57, 0.96]
TRAMETINIB	Yes	12	0.59	0.17	[0.37, 0.80]	0.33	0.19	[0.10, 0.57]

Per-drug performance for PDX-C dataset

Name	GDSC drug	N	Transfer RMSE	Transfer Std.	Transfer CI 95%	No transfer RMSE	No transfer Std.	No transfer CI 95%

(R)-(+)-Etomoxir (sodium salt)	No	13	0.05	0.008	[0.04, 0.06]	0.04	0.003	[0.04, 0.05]
968	No	13	0.12	0.019	[0.09, 0.14]	0.11	0.005	[0.11, 0.12]
A-804598	No	13	0.05	0.005	[0.04, 0.05]	0.07	0.007	[0.06, 0.08]
ABIRATERONE	No	13	0.04	0.007	[0.03, 0.05]	0.05	0.006	[0.04, 0.05]
ABT-199	No	13	0.07	0.006	[0.06, 0.08]	0.08	0.011	[0.07, 0.09]
ABT-737	No	13	0.08	0.012	[0.07, 0.10]	0.08	0.004	[0.08, 0.08]
AC55649	No	13	0.06	0.006	[0.05, 0.07]	0.07	0.006	[0.06, 0.07]
AGK-2	No	13	0.04	0.001	[0.04, 0.04]	0.04	0.005	[0.03, 0.05]
ALVOCIDIB	No	13	0.11	0.009	[0.10, 0.12]	0.17	0.013	[0.15, 0.18]
AM-580	No	26	0.07	0.005	[0.06, 0.08]	0.07	0.003	[0.06, 0.07]
APICIDIN	No	13	0.12	0.010	[0.11, 0.14]	0.13	0.010	[0.12, 0.14]
AT-406	No	13	0.13	0.014	[0.11, 0.15]	0.13	0.009	[0.12, 0.14]
AT13387	No	13	0.18	0.021	[0.15, 0.21]	0.20	0.026	[0.17, 0.23]
AT406	No	13	0.13	0.009	[0.12, 0.14]	0.13	0.005	[0.12, 0.13]
AT7867	No	13	0.26	0.025	[0.23, 0.29]	0.27	0.007	[0.27, 0.28]
AZ-3146	No	13	0.07	0.011	[0.05, 0.08]	0.11	0.017	[0.09, 0.13]
AZACITIDINE	No	13	0.14	0.012	[0.12, 0.15]	0.20	0.006	[0.19, 0.20]
AZD1480	No	13	0.12	0.004	[0.11, 0.12]	0.12	0.006	[0.11, 0.13]
AZD7545	No	13	0.04	0.009	[0.03, 0.05]	0.04	0.004	[0.04, 0.05]
BAFILOMYCIN A1	No	13	0.19	0.018	[0.16, 0.21]	0.24	0.024	[0.21, 0.27]
BARASERTIB	No	13	0.06	0.007	[0.05, 0.06]	0.06	0.009	[0.05, 0.07]
BARDOXOLONE METHYL	No	13	0.17	0.015	[0.15, 0.18]	0.15	0.005	[0.15, 0.16]
BAX CHANNEL BLOCKER	No	13	0.07	0.005	[0.07, 0.08]	0.08	0.009	[0.07, 0.09]
BEC	No	13	0.05	0.005	[0.04, 0.06]	0.06	0.007	[0.05, 0.07]
BELINOSTAT	No	13	0.16	0.016	[0.14, 0.18]	0.18	0.007	[0.17, 0.19]
BEXAROTENE	No	13	0.09	0.003	[0.09, 0.10]	0.09	0.009	[0.08, 0.10]
BIBR-1532	No	13	0.04	0.008	[0.03, 0.05]	0.08	0.016	[0.06, 0.10]
BIRB-796	No	13	0.10	0.010	[0.09, 0.11]	0.09	0.010	[0.07, 0.10]
BIRINAPANT	No	13	0.19	0.016	[0.17, 0.21]	0.19	0.014	[0.18, 0.21]
BIX-01294	No	13	0.11	0.015	[0.09, 0.13]	0.16	0.013	[0.14, 0.17]
BLEBBISTATIN	No	13	0.06	0.011	[0.05, 0.07]	0.10	0.013	[0.09, 0.12]
BLEOMYCIN	No	13	0.11	0.009	[0.10, 0.12]	0.11	0.009	[0.10, 0.12]
BMS-195614	No	13	0.04	0.005	[0.04, 0.05]	0.05	0.003	[0.05, 0.05]
BMS-270394	No	13	0.05	0.011	[0.04, 0.07]	0.05	0.006	[0.04, 0.06]
BMS-345541	No	13	0.07	0.009	[0.06, 0.08]	0.07	0.004	[0.07, 0.08]
BMS-536924	No	13	0.15	0.018	[0.13, 0.18]	0.15	0.010	[0.13, 0.16]
BORTEZOMIB	No	13	0.06	0.007	[0.05, 0.07]	0.17	0.018	[0.15, 0.20]
BOSUTINIB	No	13	0.07	0.004	[0.07, 0.08]	0.07	0.003	[0.06, 0.07]
BRD 9876	No	13	0.05	0.004	[0.04, 0.05]	0.05	0.006	[0.04, 0.06]
BRD4770	No	13	0.05	0.007	[0.04, 0.06]	0.06	0.001	[0.05, 0.06]
BREFELDIN A	No	13	0.17	0.021	[0.14, 0.19]	0.16	0.012	[0.14, 0.17]
BRIVANIB	No	13	0.06	0.011	[0.05, 0.08]	0.06	0.004	[0.05, 0.06]
BYL-719	No	13	0.11	0.007	[0.10, 0.12]	0.14	0.014	[0.12, 0.16]
C6-CERAMIDE	No	13	0.05	0.005	[0.04, 0.06]	0.05	0.005	[0.05, 0.06]

CABOZANTINIB	No	13	0.06	0.006	[0.05, 0.07]	0.07	0.011	[0.06, 0.08]
CAL-101	No	13	0.07	0.008	[0.06, 0.08]	0.08	0.005	[0.08, 0.09]
CANERTINIB	No	13	0.10	0.015	[0.08, 0.12]	0.12	0.013	[0.10, 0.13]
CAY10576	No	13	0.06	0.007	[0.05, 0.07]	0.06	0.004	[0.06, 0.07]
CAY10594	No	13	0.09	0.009	[0.08, 0.10]	0.10	0.015	[0.08, 0.12]
CAY10603	No	13	0.20	0.020	[0.18, 0.23]	0.24	0.016	[0.22, 0.26]
CCT036477	No	13	0.08	0.008	[0.07, 0.09]	0.08	0.004	[0.08, 0.09]
CD-1530	No	13	0.06	0.003	[0.05, 0.06]	0.08	0.002	[0.08, 0.08]
CD-437	No	13	0.10	0.005	[0.10, 0.11]	0.11	0.003	[0.11, 0.11]
CEDIRANIB	No	13	0.10	0.005	[0.10, 0.11]	0.10	0.005	[0.09, 0.10]
CERANIB-2	No	26	0.06	0.004	[0.06, 0.07]	0.08	0.005	[0.07, 0.08]
CERULENIN	No	13	0.05	0.004	[0.05, 0.06]	0.05	0.005	[0.04, 0.06]
CH-55	No	13	0.07	0.011	[0.05, 0.08]	0.07	0.005	[0.06, 0.07]
CHIR-99021	No	13	0.05	0.011	[0.04, 0.07]	0.06	0.004	[0.05, 0.06]
CHLORAMBUCIL	No	13	0.06	0.008	[0.05, 0.07]	0.07	0.003	[0.07, 0.08]
CI-976	No	26	0.05	0.005	[0.04, 0.05]	0.05	0.002	[0.05, 0.05]
CICLOPIROX	No	13	0.09	0.015	[0.07, 0.11]	0.09	0.005	[0.08, 0.09]
CID-2858522	No	13	0.04	0.005	[0.04, 0.05]	0.05	0.004	[0.05, 0.05]
CID-5951923	No	13	0.05	0.010	[0.04, 0.06]	0.09	0.011	[0.07, 0.10]
CIMETIDINE	No	13	0.04	0.008	[0.03, 0.05]	0.08	0.009	[0.07, 0.10]
CLOFARABINE	No	13	0.16	0.019	[0.14, 0.18]	0.17	0.012	[0.16, 0.19]
CUCURBITACIN I	No	13	0.22	0.014	[0.20, 0.24]	0.23	0.012	[0.22, 0.25]
CURCUMIN	No	13	0.07	0.005	[0.06, 0.08]	0.12	0.011	[0.11, 0.14]
CYCLOPHOSPHAMIDE	No	13	0.05	0.005	[0.04, 0.06]	0.06	0.003	[0.05, 0.06]
CYCLOSPORIN A	No	13	0.06	0.003	[0.05, 0.06]	0.07	0.008	[0.06, 0.08]
DACARBAZINE	No	13	0.04	0.004	[0.04, 0.05]	0.05	0.005	[0.04, 0.06]
DAPORINAD	No	13	0.24	0.021	[0.22, 0.27]	0.36	0.024	[0.33, 0.39]
DARINAPARSIN	No	13	0.06	0.006	[0.05, 0.06]	0.08	0.004	[0.08, 0.09]
DASATINIB	No	13	0.17	0.010	[0.15, 0.18]	0.15	0.008	[0.14, 0.16]
DBEQ	No	13	0.10	0.008	[0.09, 0.11]	0.11	0.012	[0.10, 0.13]
DECITABINE	No	13	0.06	0.021	[0.04, 0.09]	0.11	0.005	[0.10, 0.11]
DEXAMETHASONE	No	13	0.06	0.007	[0.05, 0.07]	0.06	0.005	[0.05, 0.06]
DINACICLIB	No	13	0.15	0.013	[0.13, 0.17]	0.29	0.040	[0.24, 0.34]
DNMDP	No	13	0.04	0.005	[0.04, 0.05]	0.05	0.006	[0.04, 0.05]
DOCETAXEL	No	13	0.06	0.012	[0.05, 0.07]	0.09	0.010	[0.07, 0.10]
DOXORUBICIN	No	13	0.14	0.012	[0.12, 0.15]	0.15	0.005	[0.14, 0.16]
ELOCALCITOL	No	13	0.13	0.012	[0.12, 0.14]	0.12	0.004	[0.12, 0.13]
EPIGALLOCATECHIN GALLATE	No	13	0.04	0.011	[0.02, 0.05]	0.08	0.007	[0.07, 0.08]
ERASTIN	No	13	0.04	0.003	[0.03, 0.04]	0.04	0.005	[0.03, 0.04]
ERLOTINIB	No	13	0.09	0.010	[0.08, 0.11]	0.14	0.007	[0.13, 0.15]
ETOMOXIR	No	13	0.07	0.007	[0.06, 0.07]	0.08	0.004	[0.08, 0.09]
ETOPOSIDE	No	13	0.07	0.003	[0.06, 0.07]	0.09	0.005	[0.08, 0.09]
EX-527	No	13	0.04	0.005	[0.03, 0.05]	0.04	0.006	[0.03, 0.05]
FGIN-1-27	No	13	0.05	0.005	[0.04, 0.05]	0.05	0.006	[0.04, 0.05]
FINGOLIMOD	No	13	0.09	0.017	[0.07, 0.11]	0.16	0.025	[0.13, 0.19]
FLUOROURACIL	No	13	0.05	0.009	[0.04, 0.07]	0.06	0.006	[0.05, 0.07]

FLUVASTATIN SODIUM	No	13	0.14	0.018	[0.12, 0.16]	0.12	0.005	[0.12, 0.13]
FORETINIB	No	13	0.09	0.011	[0.08, 0.11]	0.13	0.013	[0.11, 0.14]
FSC231	No	13	0.05	0.008	[0.04, 0.06]	0.07	0.009	[0.06, 0.08]
FUMONISIN B1	No	26	0.05	0.001	[0.05, 0.05]	0.05	0.001	[0.05, 0.05]
GANT 61	No	13	0.05	0.006	[0.04, 0.06]	0.05	0.002	[0.04, 0.05]
GDC-0879	No	13	0.07	0.003	[0.06, 0.07]	0.09	0.006	[0.08, 0.10]
GDC-0941	No	13	0.17	0.010	[0.16, 0.19]	0.18	0.009	[0.17, 0.19]
GMX-1778	No	13	0.24	0.031	[0.20, 0.28]	0.40	0.051	[0.34, 0.46]
GOSSYPOL	No	13	0.07	0.011	[0.05, 0.08]	0.08	0.006	[0.07, 0.09]
GSK-J4	No	13	0.10	0.013	[0.08, 0.12]	0.14	0.016	[0.12, 0.16]
GSK1059615	No	13	0.04	0.002	[0.04, 0.05]	0.05	0.002	[0.04, 0.05]
GSK2636771	No	13	0.07	0.004	[0.06, 0.07]	0.07	0.005	[0.06, 0.07]
GSK4112	No	13	0.03	0.007	[0.02, 0.04]	0.04	0.003	[0.04, 0.05]
GSK461364	No	13	0.07	0.003	[0.07, 0.07]	0.08	0.006	[0.07, 0.09]
GSK525762A	No	13	0.28	0.016	[0.26, 0.30]	0.29	0.010	[0.28, 0.30]
GW-405833	No	26	0.06	0.006	[0.05, 0.07]	0.06	0.004	[0.06, 0.07]
GW-843682X	No	13	0.06	0.007	[0.06, 0.07]	0.06	0.002	[0.05, 0.06]
HBX-41108	No	13	0.12	0.008	[0.11, 0.13]	0.12	0.001	[0.11, 0.12]
HC-067047	No	13	0.12	0.015	[0.11, 0.14]	0.13	0.004	[0.12, 0.13]
HLI 373	No	13	0.05	0.005	[0.04, 0.05]	0.05	0.003	[0.04, 0.05]
HMN-214	No	26	0.06	0.005	[0.05, 0.06]	0.07	0.003	[0.07, 0.08]
HOMOHARRINGTONINE	No	13	0.12	0.014	[0.10, 0.14]	0.23	0.023	[0.20, 0.26]
HYPERFORIN	No	13	0.17	0.017	[0.15, 0.19]	0.16	0.005	[0.15, 0.16]
I-BET151	No	13	0.32	0.021	[0.30, 0.35]	0.31	0.012	[0.30, 0.33]
IBRUTINIB	No	13	0.11	0.011	[0.09, 0.12]	0.13	0.012	[0.11, 0.14]
IC-87114	No	13	0.05	0.007	[0.04, 0.06]	0.05	0.006	[0.04, 0.06]
IFOSFAMIDE	No	13	0.05	0.007	[0.04, 0.06]	0.06	0.004	[0.05, 0.06]
IMATINIB	No	13	0.05	0.010	[0.04, 0.06]	0.04	0.002	[0.04, 0.05]
IMPORTAZOLE	No	13	0.07	0.006	[0.06, 0.08]	0.08	0.006	[0.07, 0.09]
IPR-456	No	13	0.05	0.009	[0.04, 0.06]	0.05	0.008	[0.04, 0.06]
ISOEVODIAMINE	No	13	0.07	0.009	[0.06, 0.08]	0.06	0.006	[0.05, 0.07]
ISOX	No	13	0.13	0.018	[0.11, 0.16]	0.17	0.011	[0.16, 0.19]
ISTRADEFYLLINE	No	13	0.04	0.010	[0.03, 0.05]	0.06	0.005	[0.06, 0.07]
ISX 9	No	26	0.09	0.004	[0.08, 0.09]	0.11	0.005	[0.10, 0.12]
ITRACONAZOLE	No	13	0.16	0.011	[0.15, 0.18]	0.14	0.008	[0.13, 0.15]
IU1	No	13	0.05	0.021	[0.02, 0.08]	0.08	0.017	[0.06, 0.10]
JNJ 26854165	No	13	0.12	0.013	[0.10, 0.13]	0.12	0.007	[0.11, 0.12]
JW-480	No	13	0.07	0.010	[0.06, 0.08]	0.05	0.004	[0.04, 0.05]
JW-55	No	26	0.05	0.007	[0.04, 0.05]	0.05	0.005	[0.04, 0.05]
JW-74	No	13	0.05	0.003	[0.05, 0.05]	0.05	0.004	[0.05, 0.06]
KH-CB19	No	13	0.05	0.006	[0.04, 0.06]	0.08	0.006	[0.07, 0.09]
KHS101	No	13	0.16	0.017	[0.14, 0.18]	0.17	0.011	[0.16, 0.18]
KI8751	No	13	0.06	0.003	[0.05, 0.06]	0.04	0.005	[0.04, 0.05]
KO-143	No	26	0.07	0.007	[0.06, 0.07]	0.05	0.004	[0.05, 0.06]
KU 0060648	No	13	0.06	0.007	[0.05, 0.07]	0.07	0.006	[0.06, 0.08]

KU-0063794	No	13	0.10	0.019	[0.08, 0.12]	0.18	0.019	[0.15, 0.20]
KU-60019	No	13	0.05	0.005	[0.05, 0.06]	0.05	0.006	[0.05, 0.06]
KW-2449	No	13	0.08	0.004	[0.08, 0.09]	0.09	0.004	[0.08, 0.09]
KX2-391	No	13	0.13	0.010	[0.12, 0.14]	0.14	0.014	[0.13, 0.16]
L-685458	No	13	0.04	0.001	[0.04, 0.04]	0.09	0.010	[0.07, 0.10]
LE-135	No	13	0.05	0.005	[0.04, 0.06]	0.04	0.010	[0.03, 0.05]
LENVATINIB	No	13	0.08	0.015	[0.06, 0.09]	0.13	0.016	[0.11, 0.15]
LINIFANIB	No	13	0.09	0.008	[0.07, 0.10]	0.09	0.007	[0.08, 0.10]
LOMEGUATRIB	No	13	0.05	0.005	[0.04, 0.05]	0.06	0.005	[0.06, 0.07]
LOVASTATIN	No	13	0.14	0.015	[0.12, 0.16]	0.13	0.010	[0.11, 0.14]
LRRK2-IN-1	No	13	0.12	0.011	[0.11, 0.14]	0.12	0.005	[0.12, 0.13]
LY-2157299	No	13	0.05	0.007	[0.04, 0.06]	0.05	0.007	[0.04, 0.06]
LY-2183240	No	13	0.05	0.008	[0.04, 0.06]	0.05	0.008	[0.04, 0.07]
MANUMYCIN A	No	13	0.09	0.015	[0.07, 0.11]	0.12	0.009	[0.11, 0.13]
MARINOPYRROLE A	No	13	0.12	0.011	[0.10, 0.13]	0.12	0.008	[0.11, 0.13]
MASITINIB	No	13	0.09	0.004	[0.08, 0.09]	0.08	0.005	[0.08, 0.09]
MDIVI-1	No	13	0.06	0.007	[0.05, 0.07]	0.06	0.002	[0.06, 0.07]
METHOTREXATE	No	13	0.05	0.006	[0.04, 0.05]	0.09	0.009	[0.08, 0.10]
MG-132	No	13	0.08	0.011	[0.07, 0.10]	0.19	0.022	[0.17, 0.22]
MGCD-265	No	13	0.06	0.004	[0.05, 0.06]	0.07	0.012	[0.05, 0.08]
MI-2	No	13	0.19	0.007	[0.18, 0.20]	0.20	0.008	[0.19, 0.21]
MIRA-1	No	13	0.05	0.005	[0.04, 0.05]	0.09	0.011	[0.08, 0.11]
MITOMYCIN	No	13	0.15	0.002	[0.15, 0.16]	0.16	0.006	[0.16, 0.17]
MK-0752	No	13	0.07	0.005	[0.07, 0.08]	0.07	0.006	[0.06, 0.07]
ML006	No	13	0.04	0.006	[0.04, 0.05]	0.05	0.003	[0.04, 0.05]
ML029	No	13	0.03	0.006	[0.03, 0.04]	0.04	0.007	[0.03, 0.05]
ML031	No	13	0.04	0.006	[0.04, 0.05]	0.05	0.003	[0.04, 0.05]
ML083	No	13	0.04	0.005	[0.03, 0.05]	0.04	0.007	[0.03, 0.05]
ML162	No	13	0.25	0.022	[0.23, 0.28]	0.26	0.023	[0.23, 0.28]
ML203	No	13	0.12	0.007	[0.11, 0.13]	0.14	0.004	[0.14, 0.14]
MLN2238	No	13	0.07	0.008	[0.06, 0.08]	0.19	0.040	[0.15, 0.24]
MLN2480	No	26	0.06	0.006	[0.06, 0.07]	0.08	0.003	[0.08, 0.08]
MLN4924	No	13	0.23	0.018	[0.21, 0.26]	0.25	0.012	[0.24, 0.27]
MOMELOTINIB	No	13	0.18	0.013	[0.16, 0.20]	0.18	0.004	[0.18, 0.19]
MST-312	No	13	0.08	0.005	[0.08, 0.09]	0.08	0.003	[0.07, 0.08]
MYRICETIN	No	13	0.04	0.002	[0.04, 0.04]	0.10	0.001	[0.10, 0.10]
MYRIOCIN	No	13	0.18	0.013	[0.17, 0.20]	0.16	0.006	[0.16, 0.17]
N9-ISOPROPYLOLOMOUCINE	No	13	0.09	0.016	[0.07, 0.11]	0.10	0.020	[0.07, 0.12]
NARCICLASINE	No	13	0.11	0.016	[0.09, 0.13]	0.13	0.009	[0.11, 0.14]
NECROSTATIN-1	No	13	0.04	0.006	[0.03, 0.05]	0.06	0.010	[0.05, 0.08]
NECROSTATIN-7	No	26	0.04	0.005	[0.04, 0.05]	0.04	0.003	[0.04, 0.04]
NECROSULFONAMIDE	No	13	0.07	0.006	[0.06, 0.08]	0.07	0.006	[0.06, 0.07]
NELARABINE	No	13	0.05	0.008	[0.04, 0.06]	0.06	0.006	[0.05, 0.07]
NERATINIB	No	13	0.16	0.014	[0.14, 0.18]	0.12	0.012	[0.11, 0.14]
NICLOSAMIDE	No	13	0.04	0.004	[0.03, 0.04]	0.04	0.005	[0.03, 0.05]

NINTEDANIB	No	13	0.09	0.012	[0.08, 0.11]	0.08	0.007	[0.07, 0.09]
NSC 74859	No	13	0.05	0.004	[0.05, 0.06]	0.05	0.008	[0.04, 0.06]
NSC19630	No	13	0.08	0.007	[0.07, 0.09]	0.07	0.006	[0.06, 0.08]
NSC23766	No	13	0.05	0.004	[0.04, 0.05]	0.05	0.008	[0.04, 0.06]
NSC632839	No	13	0.08	0.012	[0.06, 0.09]	0.17	0.020	[0.15, 0.20]
NSC95397	No	26	0.07	0.005	[0.06, 0.08]	0.07	0.005	[0.07, 0.08]
NUTLIN-3	No	13	0.05	0.007	[0.04, 0.06]	0.05	0.005	[0.05, 0.06]
NVP 231	No	13	0.16	0.005	[0.16, 0.17]	0.16	0.005	[0.15, 0.16]
NVP-ADW742	No	13	0.15	0.009	[0.14, 0.16]	0.15	0.008	[0.14, 0.16]
NVP-BSK805	No	13	0.09	0.015	[0.07, 0.10]	0.07	0.007	[0.06, 0.08]
NVP-TAE684	No	13	0.06	0.003	[0.05, 0.06]	0.05	0.004	[0.05, 0.06]
OBATOCLAX	No	13	0.20	0.010	[0.18, 0.21]	0.28	0.032	[0.24, 0.32]
OLIGOMYCIN A	No	13	0.23	0.014	[0.21, 0.24]	0.23	0.023	[0.20, 0.26]
OSI-930	No	13	0.05	0.008	[0.04, 0.06]	0.06	0.007	[0.05, 0.06]
OUABAIN	No	13	0.08	0.010	[0.07, 0.09]	0.12	0.019	[0.10, 0.15]
PAC-1	No	13	0.10	0.013	[0.09, 0.12]	0.09	0.009	[0.08, 0.10]
PALMOSTATIN B	No	13	0.05	0.009	[0.04, 0.06]	0.06	0.006	[0.06, 0.07]
PARBENDAZOLE	No	13	0.05	0.011	[0.04, 0.07]	0.08	0.003	[0.08, 0.08]
PARTHENOLIDE	No	13	0.06	0.007	[0.05, 0.07]	0.12	0.007	[0.11, 0.12]
PAZOPANIB	No	13	0.07	0.011	[0.06, 0.08]	0.09	0.008	[0.08, 0.10]
PD 153035	No	13	0.11	0.008	[0.10, 0.12]	0.12	0.005	[0.11, 0.13]
PD318088	No	13	0.09	0.017	[0.07, 0.11]	0.12	0.003	[0.12, 0.12]
PDMP	No	13	0.03	0.004	[0.03, 0.04]	0.04	0.002	[0.04, 0.04]
PEVONEDISTAT	No	13	0.24	0.034	[0.20, 0.29]	0.26	0.018	[0.24, 0.28]
PF-184	No	13	0.06	0.007	[0.05, 0.07]	0.07	0.002	[0.07, 0.07]
PF-3758309	No	13	0.18	0.021	[0.15, 0.20]	0.18	0.020	[0.16, 0.20]
PF-4800567 HYDROCHLORIDE	No	13	0.11	0.005	[0.10, 0.11]	0.11	0.008	[0.10, 0.12]
PF-573228	No	13	0.09	0.005	[0.08, 0.10]	0.09	0.007	[0.08, 0.10]
PF-750	No	26	0.06	0.005	[0.05, 0.06]	0.05	0.002	[0.05, 0.05]
PHA-793887	No	13	0.10	0.014	[0.09, 0.12]	0.14	0.018	[0.12, 0.16]
PHLORETIN	No	13	0.07	0.025	[0.04, 0.10]	0.09	0.012	[0.08, 0.11]
PI-103	No	13	0.15	0.027	[0.11, 0.18]	0.17	0.012	[0.15, 0.18]
PIFITHRIN-MU	No	13	0.11	0.004	[0.10, 0.11]	0.10	0.007	[0.09, 0.10]
PIK-93	No	13	0.09	0.011	[0.07, 0.10]	0.13	0.007	[0.12, 0.14]
PITSTOP2	No	13	0.04	0.011	[0.03, 0.06]	0.06	0.006	[0.06, 0.07]
PLURIPOTIN	No	13	0.16	0.015	[0.14, 0.18]	0.14	0.007	[0.13, 0.15]
PLX-4720	No	13	0.06	0.006	[0.05, 0.06]	0.06	0.005	[0.06, 0.07]
PRIMA-1	No	26	0.08	0.007	[0.07, 0.09]	0.11	0.003	[0.10, 0.11]
PRIMA-1MET	No	13	0.05	0.011	[0.04, 0.06]	0.07	0.006	[0.07, 0.08]
PRL-3 INHIBITOR I	No	26	0.05	0.003	[0.04, 0.05]	0.05	0.002	[0.04, 0.05]
PROCARBAZINE	No	13	0.05	0.008	[0.04, 0.06]	0.10	0.023	[0.08, 0.13]
PROCHLORPERAZINE	No	13	0.10	0.011	[0.08, 0.11]	0.12	0.010	[0.11, 0.13]
PURMORPHAMINE	No	13	0.05	0.007	[0.04, 0.06]	0.06	0.005	[0.06, 0.07]
PX-12	No	26	0.06	0.005	[0.05, 0.06]	0.07	0.005	[0.06, 0.07]
PYR 41	No	13	0.06	0.010	[0.04, 0.07]	0.08	0.016	[0.06, 0.10]
PYRAZOLANTHRONE	No	13	0.04	0.009	[0.03, 0.05]	0.07	0.010	[0.06, 0.08]

QS-11	No	13	0.07	0.005	[0.06, 0.08]	0.07	0.007	[0.06, 0.08]
QUIZARTINIB	No	13	0.13	0.003	[0.13, 0.14]	0.12	0.006	[0.11, 0.13]
R428	No	13	0.07	0.007	[0.06, 0.08]	0.08	0.006	[0.07, 0.09]
RAD51 INHIBITOR B02	No	13	0.07	0.004	[0.06, 0.07]	0.13	0.014	[0.12, 0.15]
RAF265	No	13	0.10	0.013	[0.09, 0.12]	0.12	0.010	[0.11, 0.13]
REGORAFENIB	No	13	0.08	0.006	[0.07, 0.09]	0.09	0.004	[0.08, 0.09]
RG-108	No	13	0.05	0.003	[0.05, 0.06]	0.07	0.008	[0.06, 0.08]
RIGOSERTIB	No	13	0.17	0.012	[0.16, 0.19]	0.19	0.016	[0.17, 0.21]
RITA	No	13	0.07	0.009	[0.06, 0.08]	0.10	0.010	[0.09, 0.11]
RO4929097	No	13	0.10	0.011	[0.08, 0.11]	0.11	0.007	[0.10, 0.12]
RUXOLITINIB	No	13	0.07	0.009	[0.06, 0.08]	0.06	0.007	[0.06, 0.07]
SALERMIDE	No	13	0.04	0.009	[0.03, 0.05]	0.07	0.007	[0.06, 0.08]
SARACATINIB	No	13	0.15	0.008	[0.14, 0.16]	0.15	0.005	[0.15, 0.16]
SB-225002	No	13	0.11	0.004	[0.10, 0.11]	0.12	0.005	[0.11, 0.12]
SB-431542	No	13	0.06	0.008	[0.05, 0.07]	0.06	0.008	[0.05, 0.07]
SB-525334	No	13	0.09	0.001	[0.09, 0.09]	0.10	0.004	[0.10, 0.11]
SB-743921	No	13	0.08	0.009	[0.07, 0.10]	0.10	0.013	[0.08, 0.11]
SB743921	No	13	0.09	0.009	[0.07, 0.10]	0.07	0.006	[0.07, 0.08]
SCH-529074	No	13	0.08	0.009	[0.07, 0.10]	0.09	0.009	[0.08, 0.10]
SCH-79797	No	13	0.07	0.011	[0.06, 0.09]	0.09	0.009	[0.08, 0.11]
SELISISTAT	No	13	0.04	0.007	[0.03, 0.05]	0.05	0.002	[0.05, 0.05]
SELUMETINIB	No	13	0.08	0.013	[0.06, 0.09]	0.09	0.010	[0.08, 0.11]
SEMAGACESTAT	No	13	0.10	0.016	[0.08, 0.12]	0.10	0.008	[0.09, 0.11]
SEPANTRONIUM BROMIDE	No	13	0.12	0.010	[0.11, 0.13]	0.11	0.005	[0.10, 0.12]
SERDEMETAN	No	13	0.12	0.011	[0.11, 0.13]	0.13	0.007	[0.12, 0.14]
SGX-523	No	13	0.05	0.005	[0.05, 0.06]	0.04	0.002	[0.04, 0.05]
SID 26681509	No	26	0.04	0.003	[0.04, 0.04]	0.05	0.005	[0.04, 0.05]
SILDENAFIL	No	13	0.05	0.007	[0.04, 0.06]	0.05	0.009	[0.04, 0.06]
SILMITASERTIB	No	13	0.05	0.010	[0.04, 0.06]	0.06	0.008	[0.05, 0.06]
SIMVASTATIN	No	13	0.13	0.011	[0.12, 0.14]	0.14	0.008	[0.13, 0.15]
SIROLIMUS	No	13	0.13	0.019	[0.11, 0.15]	0.14	0.012	[0.12, 0.15]
SITAGLIPTIN	No	13	0.04	0.006	[0.03, 0.05]	0.03	0.006	[0.03, 0.04]
SJ-172550	No	13	0.06	0.004	[0.05, 0.06]	0.07	0.004	[0.06, 0.08]
SKI-II	No	13	0.09	0.012	[0.08, 0.11]	0.09	0.009	[0.08, 0.10]
SMER-3	No	13	0.16	0.012	[0.15, 0.18]	0.16	0.009	[0.15, 0.17]
SN-38	No	13	0.16	0.022	[0.13, 0.18]	0.15	0.004	[0.15, 0.16]
SNS-032	No	13	0.17	0.012	[0.16, 0.19]	0.17	0.008	[0.16, 0.18]
SONIDEGIB	No	13	0.05	0.008	[0.04, 0.06]	0.04	0.002	[0.04, 0.04]
SOTRASTAURIN	No	13	0.15	0.009	[0.14, 0.16]	0.15	0.005	[0.14, 0.15]
SPAUTIN-1	No	13	0.09	0.006	[0.08, 0.10]	0.10	0.007	[0.09, 0.10]
SR-8278	No	13	0.05	0.008	[0.04, 0.06]	0.06	0.008	[0.05, 0.07]
SRT-1720	No	13	0.07	0.007	[0.07, 0.08]	0.13	0.014	[0.11, 0.14]
STF-31	No	13	0.17	0.020	[0.14, 0.19]	0.17	0.010	[0.16, 0.18]
SU11274	No	13	0.10	0.010	[0.09, 0.11]	0.09	0.010	[0.08, 0.10]
SUNITINIB	No	13	0.06	0.006	[0.05, 0.07]	0.06	0.009	[0.05, 0.07]

TACROLIMUS	No	13	0.04	0.004	[0.04, 0.04]	0.04	0.005	[0.04, 0.05]
TAMATINIB	No	13	0.08	0.003	[0.08, 0.09]	0.09	0.001	[0.09, 0.09]
TANDUTINIB	No	13	0.04	0.005	[0.04, 0.05]	0.04	0.005	[0.04, 0.05]
TEMSIROLIMUS	No	13	0.12	0.012	[0.11, 0.14]	0.15	0.008	[0.14, 0.16]
TENIPOSIDE	No	13	0.13	0.010	[0.12, 0.14]	0.14	0.008	[0.13, 0.15]
TG-100-115	No	13	0.06	0.011	[0.04, 0.07]	0.05	0.005	[0.05, 0.06]
TG-101348	No	13	0.17	0.026	[0.14, 0.20]	0.19	0.032	[0.15, 0.22]
TGX-221	No	13	0.09	0.011	[0.08, 0.11]	0.10	0.005	[0.09, 0.11]
THALIDOMIDE	No	13	0.05	0.007	[0.04, 0.06]	0.12	0.005	[0.11, 0.12]
TIPIFARNIB	No	13	0.09	0.007	[0.08, 0.10]	0.07	0.005	[0.06, 0.07]
TIPIFARNIB-P1	No	13	0.13	0.006	[0.12, 0.14]	0.12	0.004	[0.12, 0.13]
TIVANTINIB	No	13	0.06	0.009	[0.05, 0.07]	0.08	0.004	[0.07, 0.08]
TIVOZANIB	No	13	0.07	0.008	[0.06, 0.08]	0.07	0.003	[0.07, 0.08]
TOPOTECAN	No	13	0.14	0.009	[0.13, 0.15]	0.16	0.007	[0.15, 0.16]
TOSEDOSTAT	No	13	0.13	0.012	[0.12, 0.15]	0.13	0.004	[0.13, 0.14]
TPCA-1	No	13	0.10	0.011	[0.08, 0.11]	0.10	0.009	[0.09, 0.11]
TRETINOIN	No	13	0.04	0.004	[0.03, 0.04]	0.07	0.004	[0.06, 0.07]
TRIFLUOPERAZINE	No	13	0.15	0.013	[0.13, 0.16]	0.18	0.011	[0.16, 0.19]
TRIPTOLIDE	No	13	0.13	0.005	[0.12, 0.13]	0.21	0.025	[0.18, 0.24]
TW-37	No	13	0.14	0.011	[0.13, 0.15]	0.16	0.013	[0.15, 0.18]
UNC 0638	No	13	0.07	0.007	[0.06, 0.07]	0.08	0.007	[0.07, 0.09]
VALDECOXIB	No	13	0.04	0.005	[0.03, 0.05]	0.04	0.002	[0.04, 0.04]
VANDETANIB	No	13	0.09	0.014	[0.07, 0.11]	0.07	0.005	[0.06, 0.07]
VELIPARIB	No	13	0.04	0.009	[0.03, 0.05]	0.04	0.003	[0.04, 0.05]
VEMURAFENIB	No	13	0.05	0.008	[0.04, 0.06]	0.03	0.003	[0.03, 0.04]
VER-155008	No	13	0.08	0.010	[0.07, 0.10]	0.11	0.010	[0.10, 0.12]
VINCRISTINE	No	13	0.16	0.015	[0.14, 0.18]	0.20	0.019	[0.18, 0.22]
VORAPAXAR	No	13	0.06	0.007	[0.05, 0.06]	0.13	0.023	[0.10, 0.15]
VU0155056	No	13	0.09	0.022	[0.06, 0.12]	0.09	0.004	[0.09, 0.10]
VX-680	No	13	0.06	0.002	[0.06, 0.06]	0.06	0.003	[0.06, 0.06]
WAY-362450	No	13	0.07	0.006	[0.06, 0.08]	0.09	0.007	[0.08, 0.10]
WP1130	No	13	0.11	0.003	[0.10, 0.11]	0.13	0.007	[0.12, 0.14]
WZ4002	No	13	0.16	0.010	[0.15, 0.17]	0.16	0.010	[0.14, 0.17]
WZ8040	No	13	0.08	0.002	[0.08, 0.08]	0.11	0.017	[0.08, 0.13]
XL765	No	13	0.05	0.010	[0.04, 0.07]	0.05	0.005	[0.05, 0.06]
YK 4-279	No	13	0.04	0.009	[0.03, 0.05]	0.04	0.004	[0.03, 0.04]
YM-155	No	13	0.11	0.011	[0.10, 0.13]	0.13	0.005	[0.12, 0.14]
ZEBULARINE	No	13	0.05	0.004	[0.05, 0.06]	0.09	0.009	[0.08, 0.11]
ZSTK474	No	13	0.07	0.008	[0.06, 0.08]	0.08	0.007	[0.07, 0.09]
AFATINIB	Yes	13	0.14	0.014	[0.12, 0.16]	0.17	0.010	[0.16, 0.19]
ALISERTIB	Yes	13	0.06	0.009	[0.05, 0.07]	0.08	0.013	[0.06, 0.10]
AXITINIB	Yes	13	0.06	0.004	[0.06, 0.07]	0.05	0.004	[0.05, 0.06]
AZD4547	Yes	13	0.09	0.008	[0.08, 0.10]	0.10	0.003	[0.09, 0.10]
AZD6482	Yes	13	0.10	0.006	[0.09, 0.11]	0.11	0.005	[0.11, 0.12]
AZD7762	Yes	13	0.26	0.017	[0.24, 0.29]	0.25	0.015	[0.23, 0.27]
AZD8055	Yes	13	0.13	0.012	[0.12, 0.15]	0.22	0.019	[0.20, 0.25]
BI-2536	Yes	13	0.17	0.023	[0.14, 0.20]	0.20	0.017	[0.17, 0.22]

BMS-754807	Yes	13	0.20	0.005	[0.19, 0.20]	0.19	0.016	[0.17, 0.21]
CISPLATIN	Yes	13	0.04	0.009	[0.03, 0.05]	0.05	0.005	[0.05, 0.06]
CRIZOTINIB	Yes	13	0.08	0.010	[0.07, 0.09]	0.08	0.003	[0.08, 0.09]
CYTARABINE	Yes	13	0.13	0.021	[0.11, 0.16]	0.18	0.018	[0.16, 0.21]
DABRAFENIB	Yes	13	0.06	0.007	[0.05, 0.07]	0.08	0.005	[0.07, 0.08]
FULVESTRANT	Yes	13	0.08	0.006	[0.07, 0.09]	0.06	0.004	[0.06, 0.07]
GEFITINIB	Yes	13	0.10	0.006	[0.10, 0.11]	0.12	0.005	[0.11, 0.13]
GEMCITABINE	Yes	13	0.18	0.014	[0.17, 0.20]	0.20	0.016	[0.18, 0.22]
KU-55933	Yes	13	0.06	0.006	[0.06, 0.07]	0.06	0.007	[0.05, 0.07]
LAPATINIB	Yes	13	0.08	0.008	[0.07, 0.09]	0.10	0.005	[0.09, 0.10]
LINSITINIB	Yes	13	0.06	0.014	[0.04, 0.07]	0.06	0.005	[0.05, 0.07]
MK-1775	Yes	13	0.13	0.016	[0.11, 0.15]	0.14	0.009	[0.12, 0.15]
MK-2206	Yes	13	0.14	0.011	[0.13, 0.15]	0.17	0.006	[0.16, 0.18]
NAVITOCLAX	Yes	13	0.07	0.008	[0.06, 0.08]	0.06	0.011	[0.05, 0.08]
NILOTINIB	Yes	13	0.04	0.005	[0.04, 0.05]	0.06	0.004	[0.05, 0.06]
OLAPARIB	Yes	13	0.07	0.005	[0.06, 0.08]	0.07	0.007	[0.07, 0.08]
OSI-027	Yes	13	0.15	0.005	[0.14, 0.15]	0.15	0.003	[0.14, 0.15]
SORAFENIB	Yes	13	0.08	0.009	[0.07, 0.09]	0.08	0.007	[0.07, 0.09]
TAMOXIFEN	Yes	13	0.13	0.015	[0.11, 0.15]	0.13	0.013	[0.12, 0.15]
TEMOZOLOMIDE	Yes	13	0.04	0.005	[0.03, 0.05]	0.05	0.003	[0.04, 0.05]
TRAMETINIB	Yes	13	0.10	0.011	[0.09, 0.11]	0.12	0.011	[0.11, 0.14]
VORINOSTAT	Yes	13	0.16	0.017	[0.14, 0.19]	0.20	0.012	[0.19, 0.21]

Per-cancer performance for tumor data

			Transfer			No transfer		
**Name**	**GDSC cancer**	**N**	**AUROC**	**Std.**	**CI 95%**	**AUROC**	**Std.**	**CI 95%**
HNSCC	No	28	0.65	0.01	[0.64, 0.66]	0.51	0.08	[0.40, 0.61]
UCS	No	6	0.60	0.00	-	0.40	0.20	[0.15, 0.65]
BLCA	Yes	6	1.00	0.00	-	0.30	0.14	[0.12, 0.48]
BRCA	Yes	326	0.79	0.00	[0.79, 0.79]	0.73	0.02	[0.70, 0.75]
CESC	Yes	6	0.42	0.05	[0.36, 0.48]	0.31	0.09	[0.20, 0.43]
LGG	Yes	9	0.90	0.00	-	0.50	0.06	[0.42, 0.58]
LIHC	Yes	70	0.89	0.01	[0.87, 0.90]	0.88	0.04	[0.83, 0.93]
PAAD	Yes	6	0.22	0.00	-	0.16	0.10	[0.03, 0.28]
SKCM	Yes	33	0.54	0.00	[0.54, 0.55]	0.40	0.02	[0.37, 0.42]
STAD	Yes	9	0.22	0.00	[0.22, 0.22]	0.19	0.16	[-0.02, 0.39]

Per-cancer performance for PDX-D dataset

			Transfer			No transfer		
**Name**	**GDSC cancer**	**N**	**AUROC**	**Std.**	**CI 95%**	**AUROC**	**Std.**	**CI 95%**
CRC	No	37	0.44	0.15	[0.25, 0.63]	0.38	0.09	[0.28, 0.49]
NSCLC	No	48	0.59	0.07	[0.50, 0.67]	0.53	0.06	[0.45, 0.60]

PDAC	No	84	0.64	0.07	[0.56, 0.73]	0.55	0.08	[0.45, 0.66]
BRCA	Yes	60	0.63	0.05	[0.57, 0.70]	0.61	0.09	[0.50, 0.72]
SKCM	Yes	27	0.72	0.12	[0.57, 0.87]	0.72	0.13	[0.56, 0.87]

Per-cancer performance for PDX-C dataset

			Transfer			No transfer		
**Name**	**GDSC cancer**	**N**	**RMSE**	**Std.**	**CI 95%**	**RMSE**	**Std.**	**CI 95%**
BRCA	Yes	4641	0.10	0.0008	[0.10, 0.10]	0.12	0.0006	[0.12, 0.12]
