# Supplementary material for: Molecular pathways enhance drug response prediction using transfer learning from cell lines to tumors and patient-derived xenografts
Source: Sci Rep. 2022 Sep 27;12:16109. doi: 10.1038/s41598-022-20646-1 (PMC9515168; doi:10.1038/s41598-022-20646-1)
Supplement: Supplementary file 2 — Supplementary Information 2. [file 41598_2022_20646_MOESM2_ESM.pdf]

Source	Reference Number	Drugs
GDSC	1	CAMPTOTHECIN
GDSC	1	VINBLASTINE
GDSC	1	CISPLATIN
GDSC	1	CYTARABINE
GDSC	1	DOCETAXEL
GDSC	1	GEFITINIB
GDSC	1	NAVITOCLAX
GDSC	1	VORINOSTAT
GDSC	1	NILOTINIB
GDSC	1	OLAPARIB
GDSC	1	AXITINIB
GDSC	1	AZD7762
GDSC	1	SB216763
GDSC	1	KU-55933
GDSC	1	AFATINIB
GDSC	1	STAUROSPORINE
GDSC	1	PLX-4720
GDSC	1	DORAMAPIMOD
GDSC	1	WEE1 INHIBITOR
GDSC	1	NUTLIN-3A (-)
GDSC	1	PD173074
GDSC	1	ZM447439
GDSC	1	ALISERTIB
GDSC	1	RO-3306
GDSC	1	MK-2206
GDSC	1	PALBOCICLIB
GDSC	1	DACTOLISIB

Source	Reference Number	Cancer type
GDSC	1	Multiple myeloma
GDSC	1	Metastatic Breast Cancer
GDSC	1	Skin Cutaneous Melanoma
GDSC	1	Bladder Urothelial Carcinoma
GDSC	1	Cervical squamous cell carcinoma and endocervical adenocarcinoma
GDSC	1	Glioblastoma multiforme
GDSC	1	Lung adenocarcinoma
GDSC	1	Lung squamous cell carcinoma
GDSC	1	Small-cell carcinoma
GDSC	1	Mesothelioma
GDSC	1	Neuroblastoma
GDSC	1	Pancreatic adenocarcinoma
GDSC	1	Esophageal carcinoma
GDSC	1	Breast invasive carcinoma
GDSC	1	Head and Neck squamous cell carcinoma
GDSC	1	Acute Myeloid Leukemia
GDSC	1	Kidney renal clear cell carcinoma
GDSC	1	Ovarian serous cystadenocarcinoma
GDSC	1	Prostate adenocarcinoma
GDSC	1	Colorectal adenocarcinoma
GDSC	1	Chronic Myelogenous Leukemia
GDSC	1	Acute lymphocytic leukemia
GDSC	1	Brain Lower Grade Glioma
GDSC	1	Thyroid carcinoma
GDSC	1	Stomach adenocarcinoma
GDSC	1	Lymphoid Neoplasm Diffuse Large B-cell Lymphoma
GDSC	1	Uterine Corpus Endometrial Carcinoma

GDSC	1	PICTILISIB
GDSC	1	AZD8055
GDSC	1	PD0325901
GDSC	1	OBATOCLAX MESYLATE
GDSC	1	DASATINIB
GDSC	1	PACLITAXEL
GDSC	1	CRIZOTINIB
GDSC	1	RAPAMYCIN
GDSC	1	SORAFENIB
GDSC	1	BI-2536
GDSC	1	IRINOTECAN
GDSC	1	BMS-536924
GDSC	1	GSK1904529A
GDSC	1	TOZASERTIB
GDSC	1	PRIMA-1MET
GDSC	1	ERLOTINIB
GDSC	1	NIRAPARIB
GDSC	1	MK-1775
GDSC	1	DINACICLIB
GDSC	1	GEMCITABINE
GDSC	1	BORTEZOMIB
GDSC	1	GSK269962A
GDSC	1	SB505124
GDSC	1	TAMOXIFEN
GDSC	1	FULVESTRANT
GDSC	1	EPZ004777
GDSC	1	DAPORINAD
GDSC	1	BMS-345541
GDSC	1	AZ960
GDSC	1	TALAZOPARIB
GDSC	1	TRAMETINIB

GDSC	1	Liver hepatocellular carcinoma
GDSC	1	Chronic lymphocytic leukemia
GDSC	1	Adrenocortical carcinoma
Tumors	2, 3, 5, 10, 11, 12	Breast invasive carcinoma
Tumors	6, 9	Skin Cutaneous Melanoma
Tumors	4, 8	Liver hepatocellular carcinoma
Tumors	4	Bladder Urothelial Carcinoma
Tumors	4	Cervical squamous cell carcinoma and endocervical adenocarcinoma
Tumors	4	Colon adenocarcinoma
Tumors	4, 7	Head and Neck squamous cell carcinoma
Tumors	4	Kidney renal papillary cell carcinoma
Tumors	4	Brain Lower Grade Glioma
Tumors	4	Lung adenocarcinoma
Tumors	4	Lung squamous cell carcinoma
Tumors	4	Mesothelioma
Tumors	4	Pancreatic adenocarcinoma
Tumors	4	Sarcoma
Tumors	4	Stomach adenocarcinoma
Tumors	4	Uterine Carcinosarcoma
PDX_D	13	Breast invasive carcinoma
PDX_D	13	Colorectal cancer
PDX_D	13	Non-small cell lung cancer
PDX_D	13	Pancreatic ductal adenocarcinoma
PDX_D	13	Skin Cutaneous Melanoma
PDX_C	14	Breast invasive carcinoma

GDSC	1	DABRAFENIB
GDSC	1	TEMOZOLOMIDE
GDSC	1	RUXOLITINIB
GDSC	1	LINSITINIB
GDSC	1	EPIRUBICIN
GDSC	1	CYCLOPHOSPHAMIDE
GDSC	1	PEVONEDISTAT
GDSC	1	SAPITINIB
GDSC	1	UPROSERTIB
GDSC	1	LCL161
GDSC	1	LAPATINIB
GDSC	1	ALPELISIB
GDSC	1	EPZ5676
GDSC	1	SCH772984
GDSC	1	IWP-2
GDSC	1	LEFLUNOMIDE
GDSC	1	ENTINOSTAT
GDSC	1	OSI-027
GDSC	1	LGK974
GDSC	1	VE-822
GDSC	1	WZ4003
GDSC	1	CZC24832
GDSC	1	AZD5582
GDSC	1	GSK2606414
GDSC	1	PCI-34051
GDSC	1	WNT-C59
GDSC	1	I-BET-762
GDSC	1	RVX-208
GDSC	1	OTX015
GDSC	1	GSK343
GDSC	1	ML323
GDSC	1	ENTOSPLETINIB
GDSC	1	PRT062607
GDSC	1	RIBOCICLIB
GDSC	1	SELUMETINIB
GDSC	1	AZD4547
GDSC	1	IBRUTINIB
GDSC	1	ZOLEDRONATE
GDSC	1	CARMUSTINE
GDSC	1	TOPOTECAN
GDSC	1	TENIPOSIDE
GDSC	1	MITOXANTRONE
GDSC	1	DACTINOMYCIN
GDSC	1	FLUDARABINE

GDSC	1	NELARABINE
GDSC	1	VINCRISTINE
GDSC	1	SABUTOCLAX
GDSC	1	LY2109761
GDSC	1	OF-1
GDSC	1	MG-132
GDSC	1	BUPARLISIB
GDSC	1	ULIXERTINIB
GDSC	1	VENETOCLAX
GDSC	1	ABT737
GDSC	1	AFURESERTIB
GDSC	1	AZD3759
GDSC	1	AZD5363
GDSC	1	AZD6738
GDSC	1	AZD8186
GDSC	1	OSIMERTINIB
GDSC	1	CEDIRANIB
GDSC	1	IPATASERTIB
GDSC	1	GDC0810
GDSC	1	GNE-317
GDSC	1	GSK2578215A
GDSC	1	I-BRD9
GDSC	1	MIRA-1
GDSC	1	NVP-ADW742
GDSC	1	P22077
GDSC	1	SAVOLITINIB
GDSC	1	UMI-77
GDSC	1	SEPANTRONIUM BROMIDE
GDSC	1	MIM1
GDSC	1	WEHI-539
GDSC	1	FORETINIB
GDSC	1	BIBR-1532
GDSC	1	MK-8776
GDSC	1	VINORELBINE
GDSC	1	VX-11E
GDSC	1	VE821
GDSC	1	AZD6482
GDSC	1	AT13148
GDSC	1	BMS-754807
GDSC	1	JQ1
Tumors	5	ANASTROZOLE
Tumors	2, 5, 11	LETROZOLE
Tumors	5	EXEMESTANE

Tumors	3, 5	TAMOXIFEN
Tumors	6, 9	DABRAFENIB
Tumors	6, 9	VEMURAFENIB
Tumors	10	EVEROLIMUS
Tumors	4, 8	SORAFENIB
Tumors	7	AFATINIB
Tumors	4	GEMCITABINE
Tumors	4	CISPLATIN
Tumors	4	PACLITAXEL
Tumors	4	FLUOROURACIL
Tumors	4	IRINOTECAN
Tumors	4	RIGOSERTIB
Tumors	4	DOCETAXEL
Tumors	4	VINCRISTINE
Tumors	4	DOXORUBICIN
Tumors	4	CYCLOPHOSPHAMIDE
Tumors	4	TEMOZOLOMIDE
Tumors	4	CARMUSTINE
Tumors	4	VINORELBINE
Tumors	4	DASATINIB
Tumors	12	TRASTUZUMAB DERUXTECAN
PDX_D	13	BGJ398
PDX_D	13	BKM120
PDX_D	13	BYL719
PDX_D	13	CGM097
PDX_D	13	HDM201
PDX_D	13	RUXOLITINIB
PDX_D	13	LEE011
PDX_D	13	BINIMETINIB
PDX_D	13	PACLITAXEL
PDX_D	13	TAMOXIFEN
PDX_D	13	TRASTUZUMAB DERUXTECAN
PDX_D	13	FLUOROURACIL
PDX_D	13	ENCORAFENIB
PDX_D	13	ERLOTINIB
PDX_D	13	INC280
PDX_D	13	LGH-447
PDX_D	13	GEMCITABINE
PDX_D	13	LGK974
PDX_D	13	TRAMETINIB
PDX_D	13	DACARBAZINE
PDX_D	13	LDE225

PDX_D	13	LDK378
PDX_C	14	GSK-J4
PDX_C	14	VER-155008
PDX_C	14	SB-225002
PDX_C	14	JW-74
PDX_C	14	MITOMYCIN
PDX_C	14	GW-405833
PDX_C	14	PIFITHRIN-MU
PDX_C	14	AM-580
PDX_C	14	CCT036477
PDX_C	14	PF-750
PDX_C	14	PROCHLORPERAZINE
PDX_C	14	KHS101
PDX_C	14	PF-4800567 HYDROCHLORIDE
PDX_C	14	MST-312
PDX_C	14	HLI 373
PDX_C	14	PX-12
PDX_C	14	CI-976
PDX_C	14	ETOMOXIR
PDX_C	14	BEC
PDX_C	14	TEMOZOLOMIDE
PDX_C	14	CHIR-99021
PDX_C	14	LINIFANIB
PDX_C	14	IFOSFAMIDE
PDX_C	14	DACARBAZINE
PDX_C	14	DEXAMETHASONE
PDX_C	14	PURMORPHAMINE
PDX_C	14	SORAFENIB
PDX_C	14	KU-0063794
PDX_C	14	MANUMYCIN A
PDX_C	14	TOPOTECAN
PDX_C	14	SRT-1720
PDX_C	14	CAY10576
PDX_C	14	VEMURAFENIB
PDX_C	14	NAVITOCLAX
PDX_C	14	ETOPOSIDE
PDX_C	14	IC-87114
PDX_C	14	SCH-79797
PDX_C	14	PYR 41
PDX_C	14	CH-55
PDX_C	14	STF-31
PDX_C	14	BRD 9876
PDX_C	14	BLEBBISTATIN

PDX_C	14	FGIN-1-27
PDX_C	14	CURCUMIN
PDX_C	14	CYCLOSPORIN A
PDX_C	14	OUABAIN
PDX_C	14	LY-2183240
PDX_C	14	HBX-41108
PDX_C	14	CD-1530
PDX_C	14	TRIPTOLIDE
PDX_C	14	JW-480
PDX_C	14	PRIMA-1
PDX_C	14	LE-135
PDX_C	14	AC55649
PDX_C	14	968
PDX_C	14	RUXOLITINIB
PDX_C	14	TIPIFARNIB-P1
PDX_C	14	VELIPARIB
PDX_C	14	TG-100-115
PDX_C	14	AFATINIB
PDX_C	14	BMS-536924
PDX_C	14	AXITINIB
PDX_C	14	GSK1059615
PDX_C	14	SARACATINIB
PDX_C	14	MGCD-265
PDX_C	14	SELUMETINIB
PDX_C	14	KI8751
PDX_C	14	NINTEDANIB
PDX_C	14	PYRAZOLANTHRONE
PDX_C	14	AZD6482
PDX_C	14	SB-525334
PDX_C	14	CANERTINIB
PDX_C	14	HMN-214
PDX_C	14	BOSUTINIB
PDX_C	14	CEDIRANIB
PDX_C	14	SN-38
PDX_C	14	MARINOPYRROLE A
PDX_C	14	KO-143
PDX_C	14	YK 4-279
PDX_C	14	ISX 9
PDX_C	14	BMS-195614
PDX_C	14	KU 0060648
PDX_C	14	SCH-529074
PDX_C	14	QS-11
PDX_C	14	MIRA-1
PDX_C	14	LRRK2-IN-1

PDX_C	14	A-804598
PDX_C	14	CID-2858522
PDX_C	14	HC-067047
PDX_C	14	NSC95397
PDX_C	14	UNC 0638
PDX_C	14	NSC23766
PDX_C	14	AGK-2
PDX_C	14	GSK4112
PDX_C	14	ERLOTINIB
PDX_C	14	SUNITINIB
PDX_C	14	DASATINIB
PDX_C	14	PHA-793887
PDX_C	14	PIK-93
PDX_C	14	GEFITINIB
PDX_C	14	XL765
PDX_C	14	LAPATINIB
PDX_C	14	QUIZARTINIB
PDX_C	14	NILOTINIB
PDX_C	14	AZD7762
PDX_C	14	PAZOPANIB
PDX_C	14	SIROLIMUS
PDX_C	14	AZD8055
PDX_C	14	AT7867
PDX_C	14	PD318088
PDX_C	14	TANDUTINIB
PDX_C	14	KU-60019
PDX_C	14	EX-527
PDX_C	14	TEMSIROLIMUS
PDX_C	14	CID-5951923
PDX_C	14	JW-55
PDX_C	14	PLURIPOTIN
PDX_C	14	BMS-270394
PDX_C	14	ERASTIN
PDX_C	14	L-685458
PDX_C	14	SID 26681509
PDX_C	14	SPAUTIN-1
PDX_C	14	FLUVASTATIN SODIUM
PDX_C	14	VALDECOXIB
PDX_C	14	JNJ 26854165
PDX_C	14	HYPERFORIN
PDX_C	14	CD-437
PDX_C	14	PRL-3 INHIBITOR I
PDX_C	14	ML029

PDX_C	14	APICIDIN
PDX_C	14	SB-431542
PDX_C	14	OBATOCLAX
PDX_C	14	VANDETANIB
PDX_C	14	VX-680
PDX_C	14	RO4929097
PDX_C	14	SEMAGACESTAT
PDX_C	14	CYTARABINE
PDX_C	14	NUTLIN-3
PDX_C	14	GEMCITABINE
PDX_C	14	AZACITIDINE
PDX_C	14	GDC-0941
PDX_C	14	SONIDEGIB
PDX_C	14	KW-2449
PDX_C	14	PD 153035
PDX_C	14	RAF265
PDX_C	14	ZSTK474
PDX_C	14	BIRB-796
PDX_C	14	OLAPARIB
PDX_C	14	MASITINIB
PDX_C	14	AZD1480
PDX_C	14	GSK525762A
PDX_C	14	CERANIB-2
PDX_C	14	(R)-(+)-Etomoxir (sodium salt)
PDX_C	14	BIX-01294
PDX_C	14	YM-155
PDX_C	14	ABT-737
PDX_C	14	NECROSTATIN-7
PDX_C	14	BEXAROTENE
PDX_C	14	CERULENIN
PDX_C	14	BREFELDIN A
PDX_C	14	FUMONISIN B1
PDX_C	14	GMX-1778
PDX_C	14	BAFILOMYCIN A1
PDX_C	14	CAY10594
PDX_C	14	MLN2238
PDX_C	14	BELINOSTAT
PDX_C	14	GDC-0879
PDX_C	14	CRIZOTINIB
PDX_C	14	NVP-ADW742
PDX_C	14	MOMELOTINIB
PDX_C	14	LY-2157299
PDX_C	14	SU11274

PDX_C	14	BRIVANIB
PDX_C	14	GSK461364
PDX_C	14	TAMATINIB
PDX_C	14	NVP-TAE684
PDX_C	14	WP1130
PDX_C	14	FORETINIB
PDX_C	14	SB-743921
PDX_C	14	KU-55933
PDX_C	14	CAL-101
PDX_C	14	BI-2536
PDX_C	14	SILMITASERTIB
PDX_C	14	EPIGALLOCATECHIN GALLATE
PDX_C	14	MLN4924
PDX_C	14	LOVASTATIN
PDX_C	14	ISOX
PDX_C	14	DOXORUBICIN
PDX_C	14	ML203
PDX_C	14	MLN2480
PDX_C	14	CUCURBITACIN I
PDX_C	14	AZD4547
PDX_C	14	TIPIFARNIB
PDX_C	14	RIGOSERTIB
PDX_C	14	SALERMIDE
PDX_C	14	TAMOXIFEN
PDX_C	14	RG-108
PDX_C	14	NVP-BSK805
PDX_C	14	TENIPOSIDE
PDX_C	14	MI-2
PDX_C	14	PEVONEDISTAT
PDX_C	14	SGX-523
PDX_C	14	BARDOXOLONE METHYL
PDX_C	14	ML031
PDX_C	14	MG-132
PDX_C	14	BARASERTIB
PDX_C	14	OSI-027
PDX_C	14	MK-0752
PDX_C	14	SNS-032
PDX_C	14	PLX-4720
PDX_C	14	TW-37
PDX_C	14	ALISERTIB
PDX_C	14	IBRUTINIB
PDX_C	14	MK-2206

PDX_C	14	MYRICETIN
PDX_C	14	CABOZANTINIB
PDX_C	14	IMATINIB
PDX_C	14	ABIRATERONE
PDX_C	14	TRAMETINIB
PDX_C	14	MYRIOCIN
PDX_C	14	DOCETAXEL
PDX_C	14	KX2-391
PDX_C	14	AZ-3146
PDX_C	14	SEPANTRONIUM BROMIDE
PDX_C	14	ALVOCIDIB
PDX_C	14	SELISISTAT
PDX_C	14	GSK2636771
PDX_C	14	LINSITINIB
PDX_C	14	SERDEMETAN
PDX_C	14	DABRAFENIB
PDX_C	14	NSC 74859
PDX_C	14	ABT-199
PDX_C	14	VINCRISTINE
PDX_C	14	SOTRASTAURIN
PDX_C	14	BYL-719
PDX_C	14	SB743921
PDX_C	14	BMS-754807
PDX_C	14	AT406
PDX_C	14	SKI-II
PDX_C	14	DINACICLIB
PDX_C	14	TGX-221
PDX_C	14	PF-3758309
PDX_C	14	WZ4002
PDX_C	14	BIBR-1532
PDX_C	14	FULVESTRANT
PDX_C	14	TG-101348
PDX_C	14	AT-406
PDX_C	14	LENVATINIB
PDX_C	14	ISTRADEFYLLINE
PDX_C	14	DAPORINAD
PDX_C	14	R428
PDX_C	14	REGORAFENIB
PDX_C	14	WZ8040
PDX_C	14	TACROLIMUS
PDX_C	14	AT13387
PDX_C	14	CISPLATIN
PDX_C	14	SITAGLIPTIN

PDX_C	14	CIMETIDINE
PDX_C	14	BIRINAPANT
PDX_C	14	NARCICLASINE
PDX_C	14	PDMP
PDX_C	14	BORTEZOMIB
PDX_C	14	HOMOHARRINGTONINE
PDX_C	14	CAY10603
PDX_C	14	AZD7545
PDX_C	14	BRD4770
PDX_C	14	ML162
PDX_C	14	OLIGOMYCIN A
PDX_C	14	NECROSULFONAMIDE
PDX_C	14	NERATINIB
PDX_C	14	C6-CERAMIDE
PDX_C	14	SILDENAFIL
PDX_C	14	THALIDOMIDE
PDX_C	14	METHOTREXATE
PDX_C	14	DECITABINE
PDX_C	14	TIVOZANIB
PDX_C	14	SIMVASTATIN
PDX_C	14	BAX CHANNEL BLOCKER
PDX_C	14	CLOFARABINE
PDX_C	14	OSI-930
PDX_C	14	RITA
PDX_C	14	NICLOSAMIDE
PDX_C	14	NSC19630
PDX_C	14	GOSSYPOL
PDX_C	14	TPCA-1
PDX_C	14	VORINOSTAT
PDX_C	14	FLUOROURACIL
PDX_C	14	NELARABINE
PDX_C	14	BLEOMYCIN
PDX_C	14	ZEBULARINE
PDX_C	14	PF-573228
PDX_C	14	PAC-1
PDX_C	14	NSC632839
PDX_C	14	PF-184
PDX_C	14	TRIFLUOPERAZINE
PDX_C	14	PARTHENOLIDE
PDX_C	14	GW-843682X
PDX_C	14	GANT 61
PDX_C	14	TOSEDOSTAT

PDX_C	14	PRIMA-1MET
PDX_C	14	IU1
PDX_C	14	SMER-3
PDX_C	14	PI-103
PDX_C	14	SJ-172550
PDX_C	14	NVP 231
PDX_C	14	MDIVI-1
PDX_C	14	LOMEGUATRIB
PDX_C	14	DARINAPARSIN
PDX_C	14	MK-1775
PDX_C	14	IPR-456
PDX_C	14	DBEQ
PDX_C	14	SR-8278
PDX_C	14	I-BET151
PDX_C	14	BMS-345541
PDX_C	14	VU0155056
PDX_C	14	ELOCALCITOL
PDX_C	14	VORAPAXAR
PDX_C	14	RAD51 INHIBITOR B02
PDX_C	14	PALMOSTATIN B
PDX_C	14	KH-CB19
PDX_C	14	FSC231
PDX_C	14	TIVANTINIB
PDX_C	14	WAY-362450
PDX_C	14	ML006
PDX_C	14	PROCARBAZINE
PDX_C	14	FINGOLIMOD
PDX_C	14	CICLOPIROX
PDX_C	14	DNMDP
PDX_C	14	N9-ISOPROPYLOLOMOUCINE
PDX_C	14	PITSTOP2
PDX_C	14	NECROSTATIN-1
PDX_C	14	TRETINOIN
PDX_C	14	ISOEVODIAMINE
PDX_C	14	ITRACONAZOLE
PDX_C	14	PHLORETIN
PDX_C	14	CHLORAMBUCIL
PDX_C	14	ML083
PDX_C	14	IMPORTAZOLE
PDX_C	14	PARBENDAZOLE
PDX_C	14	CYCLOPHOSPHAMIDE
