# Supplementary material for: Molecular pathways enhance drug response prediction using transfer learning from cell lines to tumors and patient-derived xenografts
Source: Sci Rep. 2022 Sep 27;12:16109. doi: 10.1038/s41598-022-20646-1 (PMC9515168; doi:10.1038/s41598-022-20646-1)
Supplement: Supplementary file 3 — Supplementary Information 3. [file 41598_2022_20646_MOESM3_ESM.pdf]

Molecular Pathways Enhance Drug Response Prediction using Transfer Learning from Cell Lines to Tumors and Patient-Derived Xenografts

Yi-Ching Tang[1], Reid T. Powell[2], Assaf Gottlieb[1]

[1] Center for precision health, School of Biomedical informatics, University of Texas Health Science Center at Houston, Houston, TX, 77030
[2] Center for Translational Cancer Research, Texas A&M University, Houston, TX, 77030

Supplementary Materials

Supplementary Tables

Table S1. Data sources

Type	Reference Number	Sample size	Sequence platform	Gene expression identifier	Drug response measures
Cell lines	1	98,557	Array	E-MTAB-3610	AUC
Tumors	2	13	Array	GSE55374	A reduction of greater than 70% in tumor volume by 3 months
	3	55	Array	GSE16391	RFS
	4	72	RNAseq	NA	RECIST
	5	97	Array	GSE41994	TTP
	6	12	Array	GSE99898	PFS
	7	19	Array	GSE101491	RECIST
	8	67	Array	GSE109211	RFS
	9	21	Array	GSE50509	RECIST
	10	88	Array	GSE119262	Reductions in proliferation of Ki7 antibody
	11	25	Array	GSE59515	RECIST
	12	48	Array	GSE76360	RECIST
PDX-D	13	1,692	RNAseq	NA	RECIST
PDX-C	14	4,641	RNAseq	NA	AUC

Table S2. List of drug and cancer types used in the study, available as a separate file.

Table S3. Performance metrices for the classification task. Area Under the Receiver Operating Curve (AUROC) scores are averaged across 25 test folds of five repeats five-fold cross validation. The best performing model of each data is shown in bold. P-values are between Transfer and No Transfer learning schemes (Mann-Whitney U test).

		Transfer			No transfer			
Dataset	Pathway	AUROC	Std.	CI 95%	AUROC	Std.	CI 95%	P-value
Tumors	PID	0.70	0.08	[0.67, 0.73]	0.62	0.06	[0.60, 0.65]	0.001
	REACTOME	0.75	0.03	[0.74, 0.77]	0.7	0.07	[0.67, 0.73]	0.008
	PID + REACTOME	**0.77**	**0.06**	**[0.75, 0.80]**	0.72	0.04	[0.70, 0.74]	0.002
PDX-D	PID	0.63	0.11	[0.59, 0.67]	0.59	0.08	[0.56, 0.62]	0.3
	REACTOME	0.58	0.10	[0.54, 0.62]	0.5	0.02	[0.49, 0.51]	0.001
	PID + REACTOME	**0.64**	**0.08**	**[0.61, 0.68]**	0.65	0.08	[0.62, 0.68]	0.7

Table S4. Performance metrices for the regression task. Root Mean Squared Error (RMSE) scores are averaged across 25 test folds of five repeats five-fold cross validation. The best performing model of each data is shown in bold.

		Transfer			No transfer			
Dataset	Pathway	RMSE	Std.	CI 95%	RMSE	Std.	CI 95%	P-value
PDX-C	PID	**0.1**	**0.005**	**[0.10, 0.11]**	0.12	0.01	[0.12, 0.12]	$2E^{-9}$
	REACTOME	0.11	0.01	[0.10, 0.11]	0.11	0.00	[0.11, 0.11]	0.06
	PID + REACTOME	0.11	0.01	[0.11, 0.11]	0.11	0.00	[0.11, 0.11]	0.3

Table S5. Per-drug and per-cancer performance for tumor, PDX_D and PDX_C data. Available as a separate file.

Table S6. Pathway enrichment of top 10 contributing pathways for everolimus-sensitive and everolimus-resistant samples.

**Pathway**	**q-value**
INFECTIOUS DISEASE	E^{-5}
ANTI INFLAMMATORY RESPONSE FAVOURING LEISHMANIA PARASITE INFECTION	E^{-4}
ADORA2B MEDIATED ANTI INFLAMMATORY CYTOKINES PRODUCTION	$2E^{-4}$
VESICLE MEDIATED TRANSPORT	$6E^{-4}$
DEGRADATION OF THE EXTRACELLULAR MATRIX	0.004
CHEMOKINE RECEPTORS BIND CHEMOKINES	0.006
CLATHRIN MEDIATED ENDOCYTOSIS	0.01
SIGNALING BY NOTCH	0.02

Table S7. Methods comparison based on area under the receiver-operator curve (AUROC) of three drugs in the PDX-D dataset. These models included also mutation and copy-number variation data. The best performance is in bold. Paclitaxel achieves better performance using only transcriptional data (Table 2).

**Drug**	**Our model, Multi-omics**	**MOLI complete, Multi-omics [1]**	**MOLI complete Pan-drug Multi-omics [1]**
**Paclitaxel**	**0.74±0.1**	**0.74**	NA
**Gemcitabine**	0.51±0.1	**0.64**	NA
**Erlotinib**	**0.83±0.2**	0.63	0.72

[1] The performance scores were adopted from Sharifi-Noghabi (2015)'s paper.

Table S8. Hyperparameter spaces explored in this study. All values in parenthesis are (minimum, maximum).

**Parameter**	**Value range**
optimizer	[adam, adamax, adadelta, rmsprop]
Activation function	[relu, elu, tanh]
Batch size	(32, 64)
Dropout	(0.0, 0.5)
Early stop	(10, 50)
Epoch	(50, 100)
Learning rate	(0.0001, 0.1)

Neuron	(32, 512)
layer	(1, 5)
Pretrained layer	[hidden1, hidden2, hidden3, hidden4]
Retrain	[True, False]

Table S9. Model parameters

Data	Feature	optimizer	Activation function	Batch size	Dropout	Early stop	Epoch
Tumor	PID	adamax	relu	42	0.2	39	82
Tumor	REACTOME	adadelta	elu	33	0.3	37	51
Tumor	PID + REACTOME	adamax	elu	35	0	12	66
PDX-D	PID	adadelta	relu	35	0.09	12	66
PDX-D	REACTOME	adamax	elu	53	0.08	48	98
PDX-D	PID + REACTOME	adam	relu	62	0.2	49	98
PDX-C	PID	adamax	tanh	46	0.16	31	99
PDX-C	REACTOME	adam	elu	46	0.16	31	99
PDX-C	PID + REACTOME	rmsprop	elu	32	0.5	10	100
**Data**	**Feature**	**Pretrained layer**	**Retrain**	**Learning rate**	**Neuron**	**layer**	
Tumor	PID	hidden1	FALSE	0.08	89	1	
Tumor	REACTOME	hidden1	TRUE	0.05	342	1	
Tumor	PID + REACTOME	hidden1	FALSE	0.04	430	1	
PDX-D	PID	hidden2	FALSE	0.03	430	2	
PDX-D	REACTOME	hidden1	FALSE	0.09	39	2	
PDX-D	PID + REACTOME	hidden2	FALSE	0.08	217	2	
PDX-C	PID	hidden1	TRUE	0.001	281	2	
PDX-C	REACTOME	hidden3	TRUE	0.001	281	2	
PDX-C	PID + REACTOME	hidden1	TRUE	0.0001	159	2	

Supplementary Figures

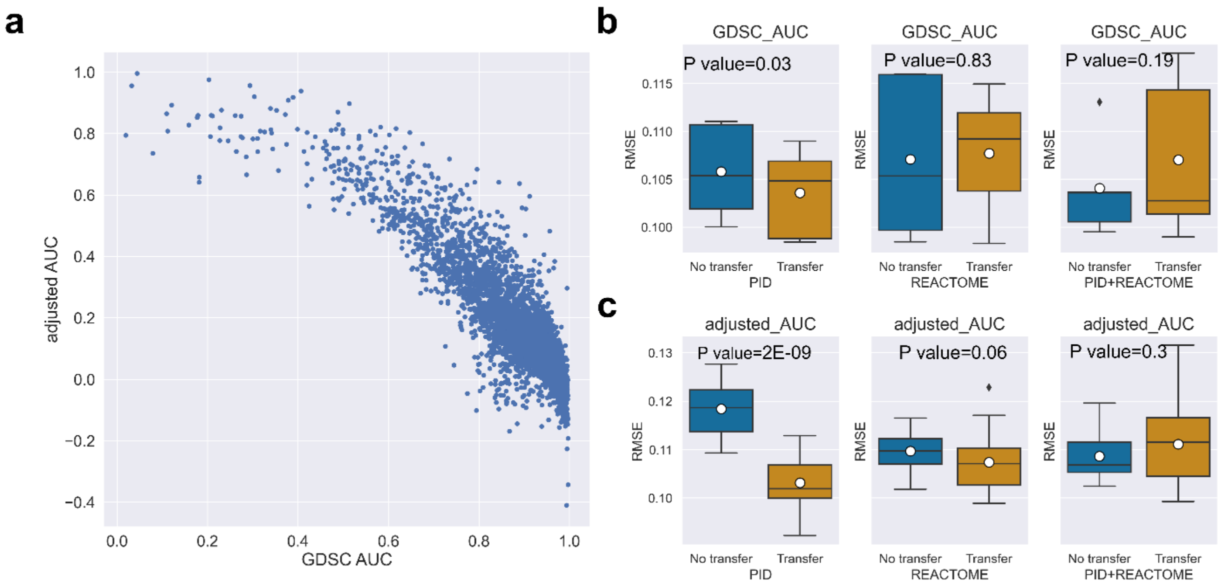

Fig. S1. Comparison of GDSC drug AUC values and adjusted AUC values. **a**. scatter plot of original AUC values in GDSC (x-axis) and adjusted AUC values (y-axis) used in previous study (Powell 2020). **b**. Box plot of Root Mean Squared Error (RMSE) of model trained with original AUC values across three pathway settings. **c**. Box plot of Root Mean Squared Error (RMSE) of model trained with adjusted AUCs.

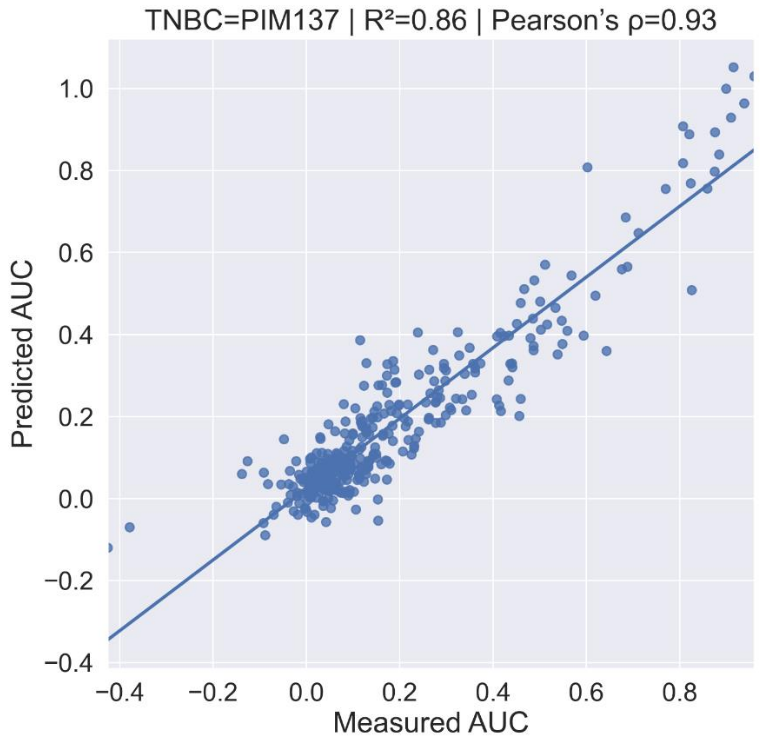

Fig. S2. Scatter plot of measured drug response AUC values (x-axis) and predicted AUC values (y-axis) of the triple-negative breast cancer (TNBC) patient-derived xenograft model.

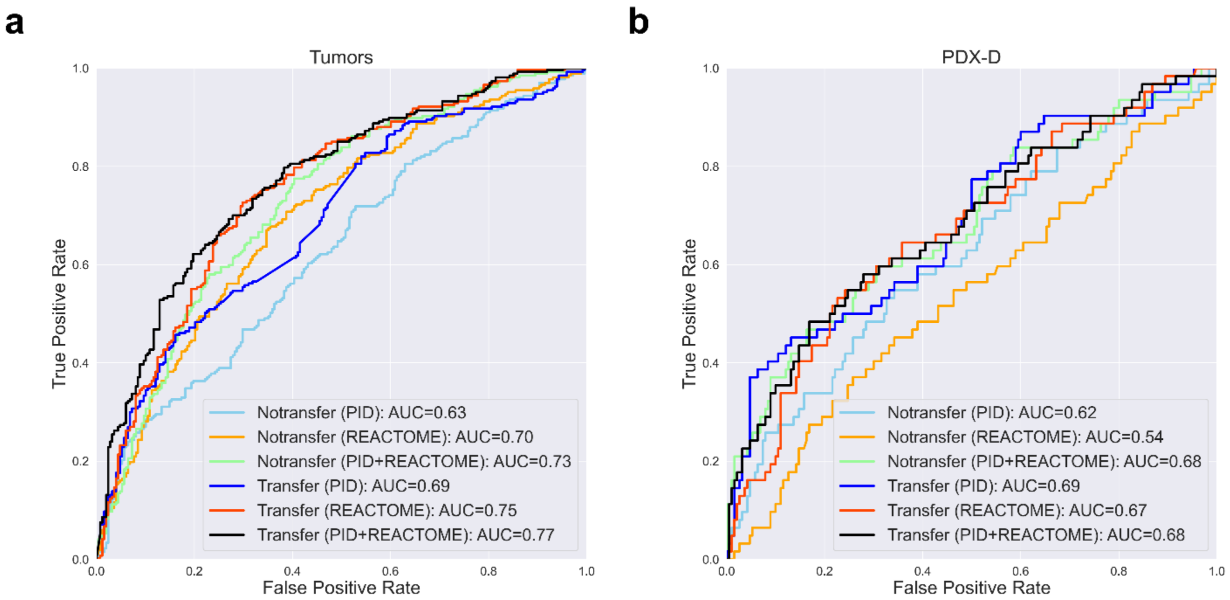

Fig. S3. Area Under the Receive Operating Curve (AUROC) of transfer learning model across different pathway datasets (PID: blue line, REACTOME: red line, both: black line) and un-transferred model from PID pathways (light blue), REACTOME (orange), and both (light green). **a**. AUROC plot for the tumor data. **b**. AUROC plot for the PDX-D dataset.

References

1. Yang, W. *et al.* Genomics of Drug Sensitivity in Cancer (GDSC): a resource for therapeutic biomarker discovery in cancer cells. *Nucleic Acids Res.* **41**, 955–961 (2012).
2. Arthur, L. M. *et al.* Molecular changes in lobular breast cancers in response to endocrine therapy. *Cancer Res.* **74**, 5371–5376 (2014).
3. Desmedt, C. *et al.* The Gene expression Grade Index: a potential predictor of relapse for endocrine-treated breast cancer patients in the BIG 1-98 trial. *BMC Med. Genomics* **2**, 40 (2009).
4. Ding, Z., Zu, S. & Gu, J. Evaluating the molecule-based prediction of clinical drug responses in cancer. *Bioinformatics* **32**, 2891–2895 (2016).
5. Jansen, M. P. H. M. *et al.* Hallmarks of aromatase inhibitor drug resistance revealed by epigenetic profiling in breast cancer. *Cancer Res.* **73**, 6632–6641 (2013).
6. Kakavand, H. *et al.* PD-L1 Expression and Immune Escape in Melanoma Resistance to MAPK Inhibitors. *Clin. Cancer Res. Off. J. Am. Assoc. Cancer Res.* **23**, 6054–6061 (2017).
7. Sittka, A. *et al.* Deep sequencing analysis of small noncoding RNA and mRNA targets of the global post-transcriptional regulator, Hfq. *PLoS Genet.* **4**, e1000163 (2008).
8. Pinyol, R. *et al.* Molecular predictors of prevention of recurrence in HCC with sorafenib as adjuvant treatment and prognostic factors in the phase 3 STORM trial. *Gut* **68**, 1065–1075 (2019).
9. Gopal, Y. N. V. *et al.* Inhibition of mTORC1/2 overcomes resistance to MAPK pathway inhibitors mediated by PGC1α and oxidative phosphorylation in melanoma. *Cancer Res.* **74**, 7037–7047 (2014).
10. Sabine, V. S. *et al.* Gene expression profiling of response to mTOR inhibitor everolimus in pre-operatively treated post-menopausal women with oestrogen receptor-positive breast cancer. *Breast Cancer Res. Treat.* **122**, 419–428 (2010).
11. Zimmerli, L. *et al.* The xenobiotic beta-aminobutyric acid enhances Arabidopsis thermotolerance. *Plant J. Cell Mol. Biol.* **53**, 144–156 (2008).
12. Varadan, V. *et al.* Immune Signatures Following Single Dose Trastuzumab Predict Pathologic Response to PreoperativeTrastuzumab and Chemotherapy in HER2-Positive Early Breast Cancer. *Clin. Cancer Res. Off. J. Am. Assoc. Cancer Res.* **22**, 3249–3259 (2016).
13. Gao, H. *et al.* High-throughput screening using patient-derived tumor xenografts to predict clinical trial drug response. *Nat. Med.* **21**, 1318–1325 (2015).
14. Powell, R. T. *et al.* Pharmacologic profiling of patient-derived xenograft models of primary treatment-naïve triple-negative breast cancer. *Sci. Rep.* **10**, 17899 (2020).
